# Single cell analysis of host response to helminth infection reveals the clonal breadth, heterogeneity, and tissue-specific programming of the responding CD4^+^ T cell repertoire

**DOI:** 10.1371/journal.ppat.1009602

**Published:** 2021-06-09

**Authors:** Ivy K. Brown, Nathan Dyjack, Mindy M. Miller, Harsha Krovi, Cydney Rios, Rachel Woolaver, Laura Harmacek, Ting-Hui Tu, Brian P. O’Connor, Thomas Danhorn, Brian Vestal, Laurent Gapin, Clemencia Pinilla, Max A. Seibold, James Scott-Browne, Radleigh G. Santos, R. Lee Reinhardt

**Affiliations:** 1 Department of Immunology and Genomic Medicine, National Jewish Health, Denver, Colorado, United States of America; 2 Center for Genes, Environment, and Health, National Jewish Health, Denver, Colorado, United States of America; 3 Department of Immunology and Microbiology, University of Colorado Anschutz Medical Campus, Aurora, Colorado, United States of America; 4 Department of Pediatrics, National Jewish Health, Denver, Colorado, United States of America; 5 Florida International University, Port Saint Lucie, Florida, United States of America; 6 Division of Pulmonary Sciences and Critical Care Medicine, University of Colorado School of Medicine, Aurora, Colorado, United States of America; 7 Department of Mathematics, Nova Southeastern University, Fort Lauderdale, Florida, United States of America; University of Medicine & Dentistry New Jersey, UNITED STATES

## Abstract

The CD4^+^ T cell response is critical to host protection against helminth infection. How this response varies across different hosts and tissues remains an important gap in our understanding. Using IL-4-reporter mice to identify responding CD4^+^ T cells to *Nippostrongylus brasiliensis* infection, T cell receptor sequencing paired with novel clustering algorithms revealed a broadly reactive and clonally diverse CD4^+^ T cell response. While the most prevalent clones and clonotypes exhibited some tissue selectivity, most were observed to reside in both the lung and lung-draining lymph nodes. Antigen-reactivity of the broader repertoires was predicted to be shared across both tissues and individual mice. Transcriptome, trajectory, and chromatin accessibility analysis of lung and lymph-node repertoires revealed three unique but related populations of responding IL-4^+^ CD4^+^ T cells consistent with T follicular helper, T helper 2, and a transitional population sharing similarity with both populations. The shared antigen reactivity of lymph node and lung repertoires combined with the adoption of tissue-specific gene programs allows for the pairing of cellular and humoral responses critical to the orchestration of anti-helminth immunity.

## Author summary

Using various “omic” approaches, the CD4^+^ T cell receptor (TCR) repertoire was explored after primary helminth infection. Infection generated a broadly reactive and clonally diverse CD4^+^ T cell response with the most prevalent clonotypes and predicted antigen specificities residing in both the lung and lung-draining lymph nodes. Tissue-specific programming of responding CD4^+^ T cells directed the establishment of committed Tfh and Th2 cells, both critical for driving distinct hallmarks of type-2 inflammation. These datasets help to explore the diverse yet tissue-specific nature of anti-helminth immunity.

## Introduction

CD4^+^ T helper 2 (Th2) cells have been shown to exhibit remarkable heterogeneity in gene expression and cytokine potential in settings of type-2 inflammation in both mice and humans [1–3]. Our understanding of this heterogeneity has been greatly advanced using repeated or prolonged exposure to model allergens and antigens [4–6]. While providing an important context for understanding the host response in settings of type-2 immunity, such studies are restricted to evaluating CD4+ T cells of known antigen specificity directed against dominant epitopes. As such, how the type-2 CD4+ T cell repertoire changes in response to more epitope-rich antigenic stimuli, such as that found in parasitic helminth infection, remains poorly understood. These large extracellular worms express and secrete a diverse array of stage-specific proteins which influence both the immunogenic epitopes available during infection and the tissue-specific nature of the immune response [7,8]. Consistent with the complex nature and number of antigens associated with helminth infection, antibody staining of select T cell receptor (TCR) β chains after *N*. *brasiliensis* infection suggests a diverse Th2 cell response in lymph nodes [9]. However, there remains a paucity of data investigating the breadth, specificity, and heterogeneity of the CD4^+^ T cell response during such infections. Specifically, the presence of public or dominant clonotypes, breadth of antigen specificity, relatedness between TCR repertoires in lymphoid and nonlymphoid tissues of the same mouse or across different mice, and heterogeneity of the IL-4^+^ T cell compartment in response to helminth infection are not known.

Although studies investigating CD4^+^ T cells in settings of type-2 inflammation have focused on the canonical Th2 cell compartment, it is becoming increasingly clear that IL-4^+^ T follicular helper (Tfh) cells play an essential yet distinct role among the responding CD4^+^ T cell population to helminths [10–12]. CD4^+^ Th2 cells producing IL-4, IL-5, and IL-13 direct cellular immunity in mucosal tissues to maintain barrier integrity after helminth infection [13]. Lymphoid-resident CD4^+^ Tfh cells orchestrate the humoral arm of a typical type-2 immune response by helping B cells produce immunoglobulin (Ig)-E and IgG1 [14–16]. The production and optimal affinity of these isotypes largely depends on the presence of IL-4-expressing Tfh cells in the context of helminth infection and IL-13-producing Tfh cells in the context of high-affinity IgE after allergen challenge [16,17]. To mount the most productive immune response to a pathogen, cell-mediated immunity is often linked to the humoral response. The easiest way to link Th2 cell-mediated responses in nonlymphoid tissues with the antibody responses orchestrated by Tfh cells in the lymph nodes is for each of these T cell populations to exhibit similar antigen reactivity. This can be readily accomplished in two ways. First, the responding Tfh and Th2 cell repertoires can contain common clones that express the same TCRs. Second, Tfh cells and Th2 cells can express different TCRs that share specificity for the same peptide-MHC complex. In both cases, these Tfh and Th2 cells would be clustered into the same antigen specificity group based on their reactivity to an identical antigen. As such, CD4^+^ Tfh and Th2 cells sharing antigen specificity serve as an important bridge between humoral and cell-mediated immunity generated after helminth infection [18].

To better understand the pivotal role IL-4^+^ Th2 and Tfh cell subsets play in the orchestration of productive type-2 immunity against helminths, we took an unbiased approach combining the use of sensitive IL-4-reporter mice with bulk and single cell TCR-, RNA-, and ATAC-sequencing to characterize the anti-helminth CD4^+^ T cell response to *N*. *brasiliensis* infection. Two novel algorithms designed to group TCRs based on their predicted antigen specificity were used to reveal the breadth and depth of the responding TCR repertoire in the lymph nodes and lung across different animals, tissues, and strains. Single cell transcriptome and chromatin accessibility were subsequently used to identify unique gene sets, regulatory regions, and transcription factor binding sites that discriminate between IFN-gamma^+^ and IL-4^+^ CD4^+^ T cells as well as Th and Tfh subsets responding to helminth infection. Lastly, cell trajectory analysis revealed a putative IL-4^+^ precursor that could give rise to both IL-4^+^ Tfh and Th2 cells. Together, these approaches reveal the complex nature of anti-helminth immunity and highlight the tissue-specific influence on T cells in settings of type-2 immunity.

## Results

### TCRβ repertoire analysis of IL-4^+^ CD4^+^ T cells in the lung and mediastinal lymph nodes after *N*. *brasiliensis* infection

**Fig 1:**
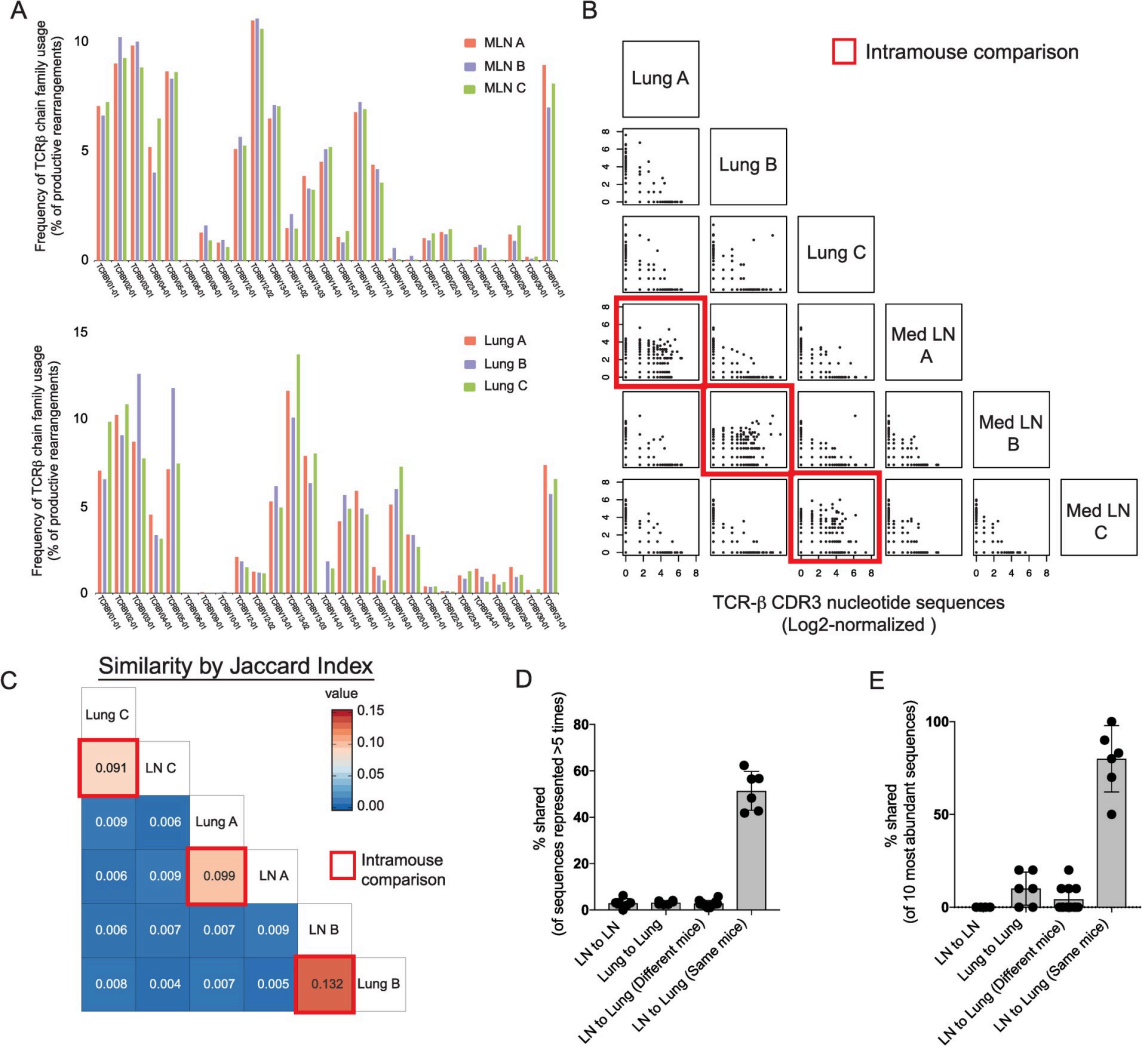
IL-4-expressing CD4^+^ T cells in the lymph node and lung share TCRβ sequences in the same mouse but rarely across different mice after *N*. *brasiliensis* infection. Three separate IL-4^4get^ C57BL/6 mice (denoted A, B, C) were infected with *N*. *brasiliensis* and GFP^+^ CD4^+^ T cells from the lung and mediastinal lymph nodes were harvested and sorted nine days post-infection for TCRβ analysis. **(A)** Bar graphs represent the percentage of TCRβ sequences from IL-4^+^ CD4^+^ T cells isolated from mediastinal lymph node (top) and the lung (bottom) of each mouse that are found in the indicated TCRβ chain families. **(B)** Diagonal scatter plots comparing Log_2_-normalized TCRβ CDR3 nucleotide sequences of IL-4-expressing CD4^+^ T cells from the mediastinal lymph nodes and lungs of indicated mice. Red boxes indicate intramouse comparisons. **(C)** Jaccard similarity coefficient matrix comparing the similarity of TCR-β sequences found in the mediastinal lymph nodes and lungs of different mice and the lymph nodes and lung of the same mouse. Red boxes indicate intramouse comparisons. **(D)** The graph represents the percentage of TCRβ sequences found at least 5 times in one tissue that are shared among TCRβ sequences found in the other tissues within the same mouse or across different mice. **(E)** Graph represents the percentage of the top 10 most abundant TCRβ sequences in each tissue that are shared among other indicated tissues; error bars represent +/- SD; n = 3 mice.

To investigate the responding CD4^+^ T cell repertoire and response to helminths, we infected IL-4^4get^ reporter mice with the helminth *N*. *brasiliensis* and performed TCRβ sequencing on sorted IL-4-expressing CD4^+^ T cells from the lung and lung-draining mediastinal lymph nodes nine days after infection. In the IL-4^4get^ mouse, all cells transcribing IL-4 mRNA will express green fluorescent protein (GFP) [19]. TCRVβ family usage was similar when comparing IL-4^+^ CD4^+^ T cells isolated from the mediastinal lymph nodes of different mice (Fig 1A). More variation was observed among IL-4^+^ CD4^+^ T cell populations residing in the lungs (Fig 1A). To assess whether the similar Vβ family usage reflected the presence of shared (public) clonotypes within these IL-4^+^ populations, TCRβ sequences were compared across tissues and mice. Pairwise scatterplots show that both unique and shared TCRβ sequences are present in the lung and lymph node of the same mouse (Fig 1B). However, when comparing tissues of different mice, few shared or public sequences were observed. Complimentary findings were obtained when comparing amino acid sequences in C57BL/6 mice and amino acid and nucleotide sequences from CD4^+^ T cells obtained from *N*. *brasiliensis*-infected BALB/c mice (S1A–S1C Fig).

To determine the similarity of the TCRβ repertoire in different mice and tissues, the Jaccard similarity index was used. This is a quantitative measure of the similarity between two populations with a higher percentage representing more similarity [20]. Percent similarity revealed that the same TCRβ sequences were significantly more likely to appear in the lung and lymph node of the same mouse than in tissues from different mice (Fig 1C). When restricting our analysis to the most prevalent TCRβ sequences, we again observed that sequences were commonly shared between the lymph node and lung of the same mouse but not across different mice (Fig 1D and 1E). We confirmed these findings in IL-4-competent CD4^+^ T cells isolated from *N*. *brasiliensis*-infected BALB/c mice (S1D and S1E Fig). The lack of shared TCRβ sequences across mice suggests that “public clonotypes” appear only rarely in response to helminth infection even among genetically similar mice.

### The most abundant IL-4-expressing clones in the lung and mediastinal lymph node after helminth infection share clonal origins

**Fig 2:**
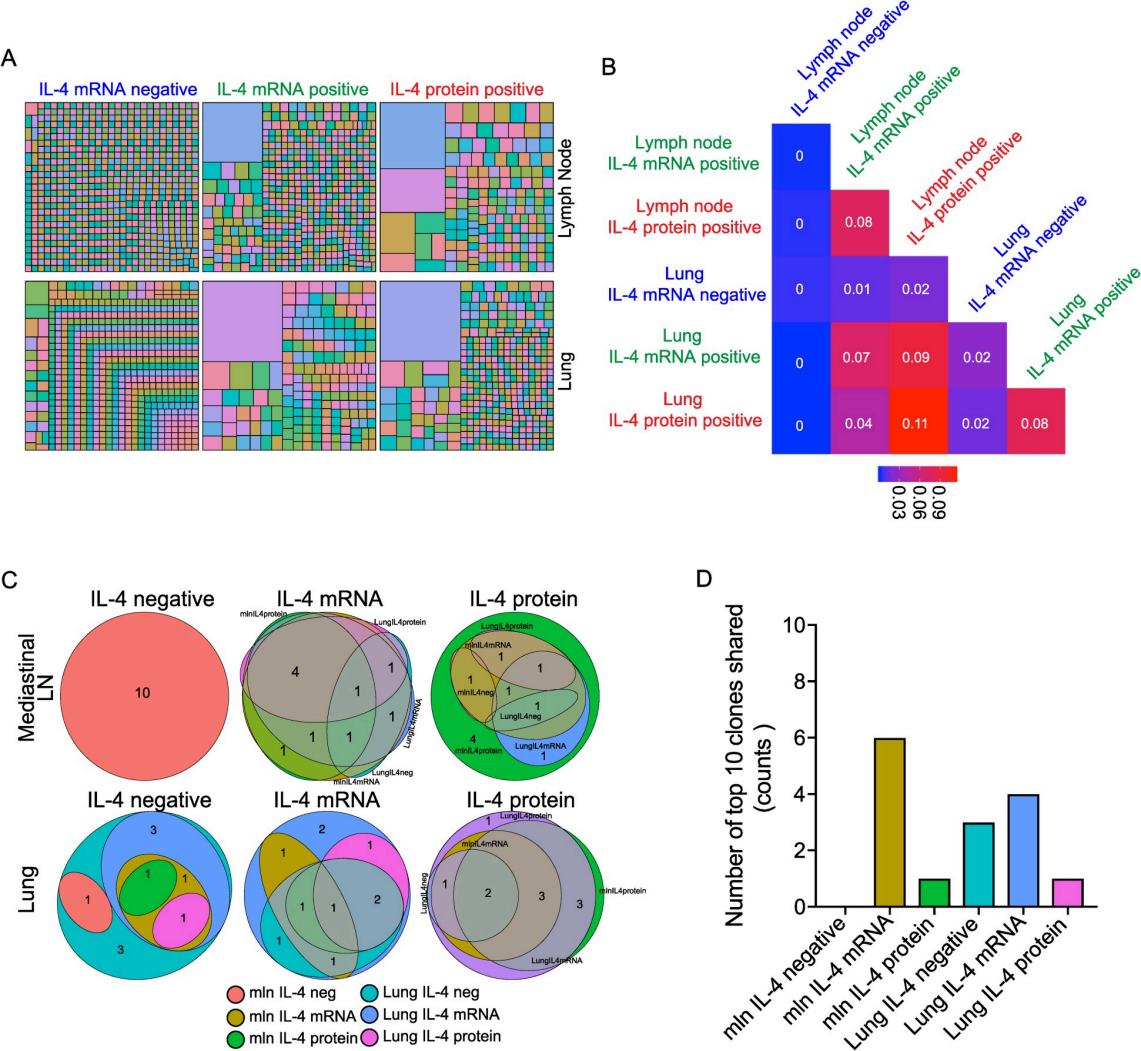
Single-cell sequencing of the T cell receptor repertoire reveals shared clonal origins among the most prevalent clonotypes in the lymph nodes and lung after helminth infection. The lung and mediastinal lymph nodes were harvested from an IL-4^4get-KN2^ C57BL/6 mouse that was infected nine days prior with *N*. *brasiliensis*. The GFP^+^ and GFP^-^ CD4^+^ T cells were sorted and single-cell sequencing of the TCRα and β chains was performed. **(A)** Tree map showing clonal abundance of the top 792 clones in each of the indicated tissues. Each clone was assigned a random color and the size of the box represents its clonal abundance among the pool of clones sequenced in the indicated tissue. **(B)** Jaccard similarity coefficient matrix comparing the similarity of TCR-α/β sequences of IL-4^+^ and IL-4^-^ CD4^+^ T cells isolated from the mediastinal lymph node and lung. **(C)** Euler plots represent the overlap of the top 10 clones observed in each group of 6 groupings of T cells; number in each oval represents the number of top 10 clones that are present in other groups. **(D)** Bar graph shows the number of top 10 clones in each group that are also a part of the top 10 clones in other T cell groupings.

The TCRβ sequencing results are consistent with IL-4-expressing CD4^+^ T cells in the lymph nodes and lungs sharing a common clonal origin. We next wanted to confirm clonality within these tissue-specific repertoires and investigate whether any tissue-specific repertoire differences existed among cells actively producing IL-4. To do this, we performed paired TCRα and TCRβ chain sequencing on single CD4^+^ T cells isolated from IL-4^4get-KN2^ reporter mice nine days after *N*. *brasiliensis* infection. In these dual cytokine-reporter mice, GFP^+^, human CD2 (huCD2)^+^ CD4^+^ T cells represent IL-4^+^ cells that have most recently encountered antigen and secreted protein (huCD2 expression marks cells with recent IL-4 protein production) [21]. In the context of helminth infection, the majority of GFP^+^, huCD2^+^ cells are restricted to the B cell follicles and by definition are Tfh cells at this timepoint [16]. Sequencing revealed that both IL-4 mRNA- and IL-4 protein-reporter positive T cells showed clonal enrichment in the mediastinal lymph node and lung (Fig 2A). Similar to Jaccard index comparisons of bulk TCRβ chain sequencing, IL-4 mRNA-expressing CD4^+^ T cells isolated from the lymph node and lung showed a high degree of relatedness (Fig 2B). Of the populations compared, the highest degree of similarity was between IL-4 protein-expressing CD4^+^ T cells in the lung and lymph node. Given that Th2 cells in the lung and Tfh cells in the lymph nodes are the major IL-4-producing populations in these distinct tissues, the sharing of IL-4-producing clones between these tissues provides a link between the antibody response (orchestrated by IL-4-producing Tfh cells) in the lymph nodes and the cell-mediated response (orchestrated by Th2 cells) in the lung. Pairing of IL-4-producing clones in the lymph nodes and lungs is likely important for the host’s ability to match the appropriate antibody specificity with the appropriate T cell specificity to maximize helminth clearance.

When the top 10 most abundant clones of each of the six groups were compared, 52 unique clonotypes were observed (S1 Table). Of the top clones in the mediastinal lymph nodes, none of the top 10 clones from the IL-4^-^ pool were found in any of the other groups, while all of the top 10 IL-4 mRNA^+^ clones and 6 of the top 10 IL-4 protein^+^ clones were found at least once in other groups (Fig 2C). When assessing the relationship between the top IL-4^+^ mRNA clones and IL-4^+^ protein clones in the mediastinal lymph node, 8 of the top IL-4 mRNA^+^ sequences were found within the IL-4 protein^+^ group in the mediastinal lymph node; 9 were shared by the IL-4 mRNA^+^ group in the lung; and 6 were found in the IL-4 protein^+^ group in the lung. This confirms that the most prevalent IL-4-competent clones in the lymph nodes also reside in the lung. However, a more restricted pattern emerged among IL-4 protein^+^ clones in the lymph nodes. Here, only 5 of the top 10 clones were observed in the IL-4 mRNA^+^ group obtained from the mediastinal lymph node, and only 4 and 2 clones were shared by the IL-4 mRNA^+^ and IL-4 protein^+^ groups in the lung, respectively. Thus, there is some selection for the clones that go on to produce IL-4 protein in the lymph node.

In contrast, when focusing on the top 10 IL-4 mRNA^+^ and IL-4 protein^+^ clones in the lung, it is the IL-4 protein^+^ group that is highly shared across groups and tissues (Fig 2C). All of the top IL-4 protein^+^ clones in the lung are found in the IL-4 mRNA^+^ group in the lung with 8 clones being shared with IL-4 protein^+^ group in the lymph nodes. As such, the IL-4 protein^+^ clones in the lung are not as restricted across lymphoid and nonlymphoid tissues as their lymph node counterparts. That said, there is still a degree of clonal preference among the top IL-4 protein producers in the lung as few of these clones are found within the top 10 clones of the other groups analyzed (Fig 2D). Clonal preference is also evident in the lymph nodes as none of the top 10 IL-4 protein^+^ clones found in the lymph node were present among the top 10 IL-4 protein^+^ clones in the lung (S1 Table). This suggests that while overall the clonal repertoires are similar across lymphoid and lung tissue, there is preferential clonal dominance indicative of tissue-specific repertoire differences. It is interesting to speculate about the enrichment of clones in these distinct tissues. Clones enriched in the lymph nodes are consistent with enrichment of Tfh cells. In contrast, lung-dominant clones may reflect tissue-resident Th2 memory cells that preferentially reside in nonlymphoid tissues and do not recirculate. While the sharing of IL-4-producing clones discussed above may allow antigen specificities of the humoral response to be matched with that of the cell-mediated response, the enrichment of certain clones in the lymph nodes compared to the lung may reflect important differences in their requirement for antigenic stimulation. For example, prolonged signaling or higher affinity for antigen may be advantageous for Tfh cells helping to select B cells in germinal centers, while less affinity for antigen may be advantageous to Th2 cells when trying to promote type-2 responses in the lung while simultaneously attempting to limit excessive inflammation.

### Predicted antigen specificity groupings are shared across mice and tissues after helminth infection

The lack of shared or public TCRβ or TCRα/β sequences made it difficult to assess whether similar antigen specificities were found across repertoires obtained from tissues of different mice. To investigate whether IL-4-expressing CD4^+^ T cells were likely responding to similar antigens across different mice, we first used the grouping of lymphocyte interactions by paratope hotspots (GLIPH) algorithm, which clusters TCRs that have related but not identical TCR sequences with a high probability of sharing the same antigen specificity [22]. GLIPH predicts antigen specificity groups using parameters focused on TCR motif conservation and similarities in length and sequence identity of complementarity-determining region 3 (CDR3). GLIPH queries the probability that a given cluster of similar TCRs could arise without the selection of a common antigen. TCRs of likely shared antigen specificity are scored and clustered into individual TCR convergence groups (CRGs).

**Fig 3:**
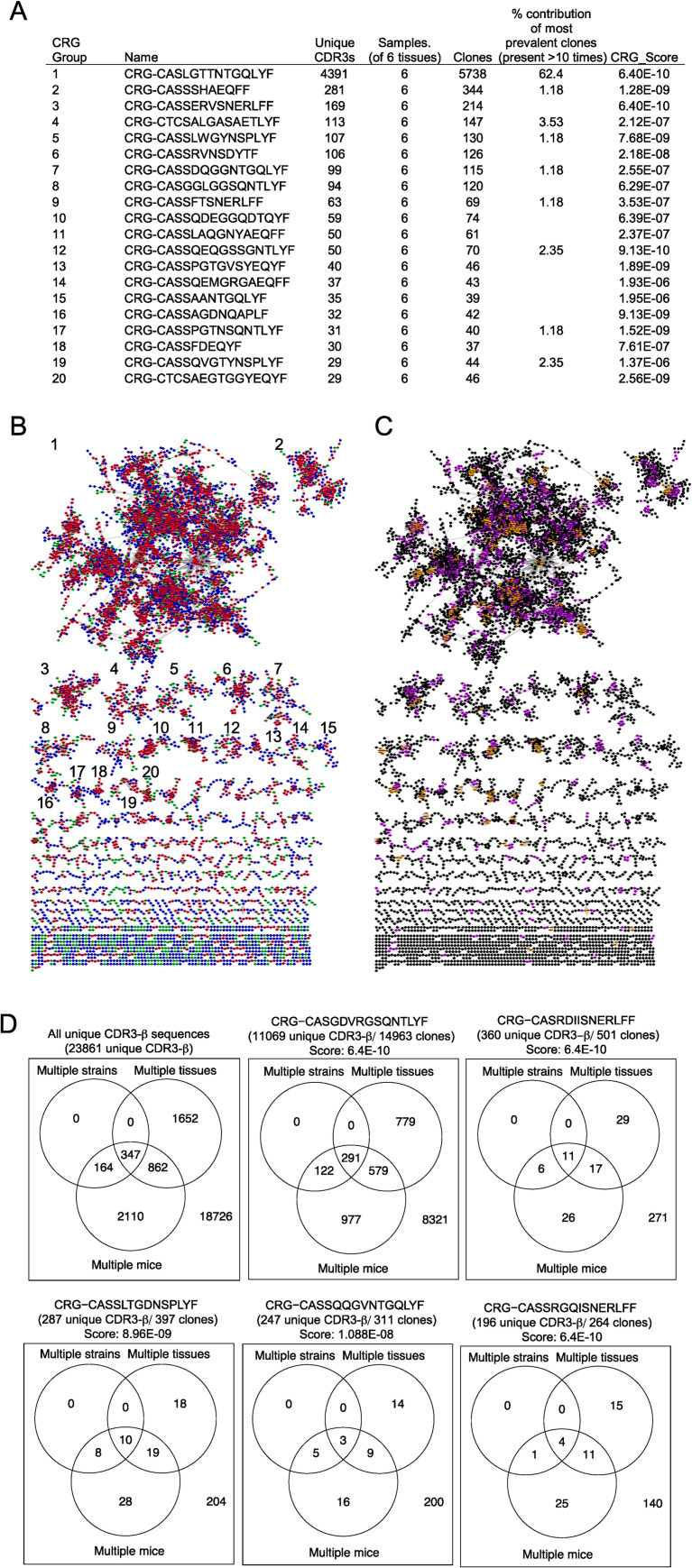
TCR convergence groups indicate shared antigen specificities across mice and tissues. GLIPH analysis was performed on IL-4^+^ CD4^+^ T cells from the mediastinal lymph nodes and lung of IL-4^4get^ mice nine days after *N*. *brasiliensis* infection. GLIPH identified 5518 convergence groups (CRGs) from 12,838 unique TCRβ sequences. **(A)** Summary of GLIPH scoring for the 20 largest convergence groups based on unique CDR3 numbers. **(B)** Network analysis of all CRGs comprised of two or more unique CDR3 regions. Numbers correspond to CRGs listed in (A). Each node represents a unique TCR. Each edge represents similarity between nodes that differ by ≤ 2 amino acids or share a significant motif. Red denotes nodes that share similarity (edge) with other nodes that are found in both the lung and lymph nodes. Green represents nodes that share similarity with other nodes found only in the lung. Blue represents nodes that share similarity with other nodes found only in the lymph nodes. **(C)** Network analysis of nodes in (B). Orange represents nodes that share similarity with other nodes found in all three mice. Purple represents nodes that are shared between two mice, and black represents nodes that share similarity only with nodes found in the same mouse. **(D)** Venn diagrams represent how unique TCRβ sequences are shared across individual mice, different strains, and tissues. The first square represents the distribution of all 23,861 TCR-β sequences isolated from IL-4^+^ T cells in both C57BL/6 and BALB/c strains after N. brasiliensis infection. The remaining 5 squares represent the breakdown of overlap between sequences found in the 5 largest GLIPH clusters.

By combining all 12,838 independent TCRβ sequences from the pool of IL-4-competent CD4^+^ T cells sequenced from three mice, GLIPH identified 5518 CRGs or predicted antigen specificity groups. Of the 5518 CRGs identified, 6 contained greater than 100 unique CDR3βs and represented 40.2% of the total TCRs sequenced (Fig 3A); 726 CRGs contained two or more unique CDR3β sequences and 38 CRGs consisted of 10 or more unique sequences. The top specificity group (CRG group 1) consisted of 4391 unique CDR3β sequences and contained 62.4% of clonotypes found greater than 10 times in any tissue or mouse. CDR3βs in this cluster were found across all 6 tissues (samples) from 3 different mice. The majority of these specificity groups consisted of TCRs that shared similarity within their CDR3βs with other TCRs found in both the lymph node and lung (Fig 3B). This is highlighted in the network analysis where each red node (a unique CDR3) shares similarity with other nodes found in both the lung and lymph node. Blue and green nodes represent CDR3s showing similarity with other nodes found in only one of the two tissues. When assessing shared similarity between CDR3s across mice, network analysis revealed that nodes in one mouse commonly shared similarity to other nodes found in different mice (Fig 3C). Purple and orange nodes represent similarities shared between 2 and 3 mice, respectively. Black nodes indicate CDR3 sequences that share similarity with other nodes of same mouse (1 mouse).

To assess if there were likely shared antigen specificity groups across strains, we also performed GLIPH analysis on CD4^+^ T cells isolated from three IL-4^4get^ BALB/c and the three IL-4^4get^ C57BL/6 mice previously infected with *N*. *brasiliensis*. As was shown across different human HLA-types using GLIPH-based algorithms, we were able to identify antigen specificity groups that harbored CDR3 regions from both strains of mice [22,23]. Of the 23,861 unique CDR3β sequences found in the three C57BL/6 and three BALB/c mice, 2,861 were found in both the lung and lymph nodes, and 3,483 were shared across multiple mice as designated by summing of values in the corresponding circles (Fig 3D). While rare, 511 (multiple strains) sequences were found in both C57BL/6 and BALB/c strains of mice. When focusing on the five largest CRG groups, all showed multiple CDR3β sequences that were shared across strains, mice, and tissues. While this highlights that public IL-4^+^ clonotypes shared across strains exist, they are very rare. In the context of the broad anti-helminth repertoire, most of the shared antigen reactivity that spans tissues, mice, and strains comes from CD4^+^ T cells that are unique to a specific tissue, mouse, and strain. Overlap of unique clonotypes in specificity groups suggests that each mouse is likely to respond somewhat similarly to the diverse antigenic load that results after infection by these large extracellular parasites, despite possessing largely unique repertoires.

### Hierarchical Clustering Appearing First increases accuracy of antigen specificity groups and clarifies consensus TCR sequences in larger clusters

**Fig 4:**
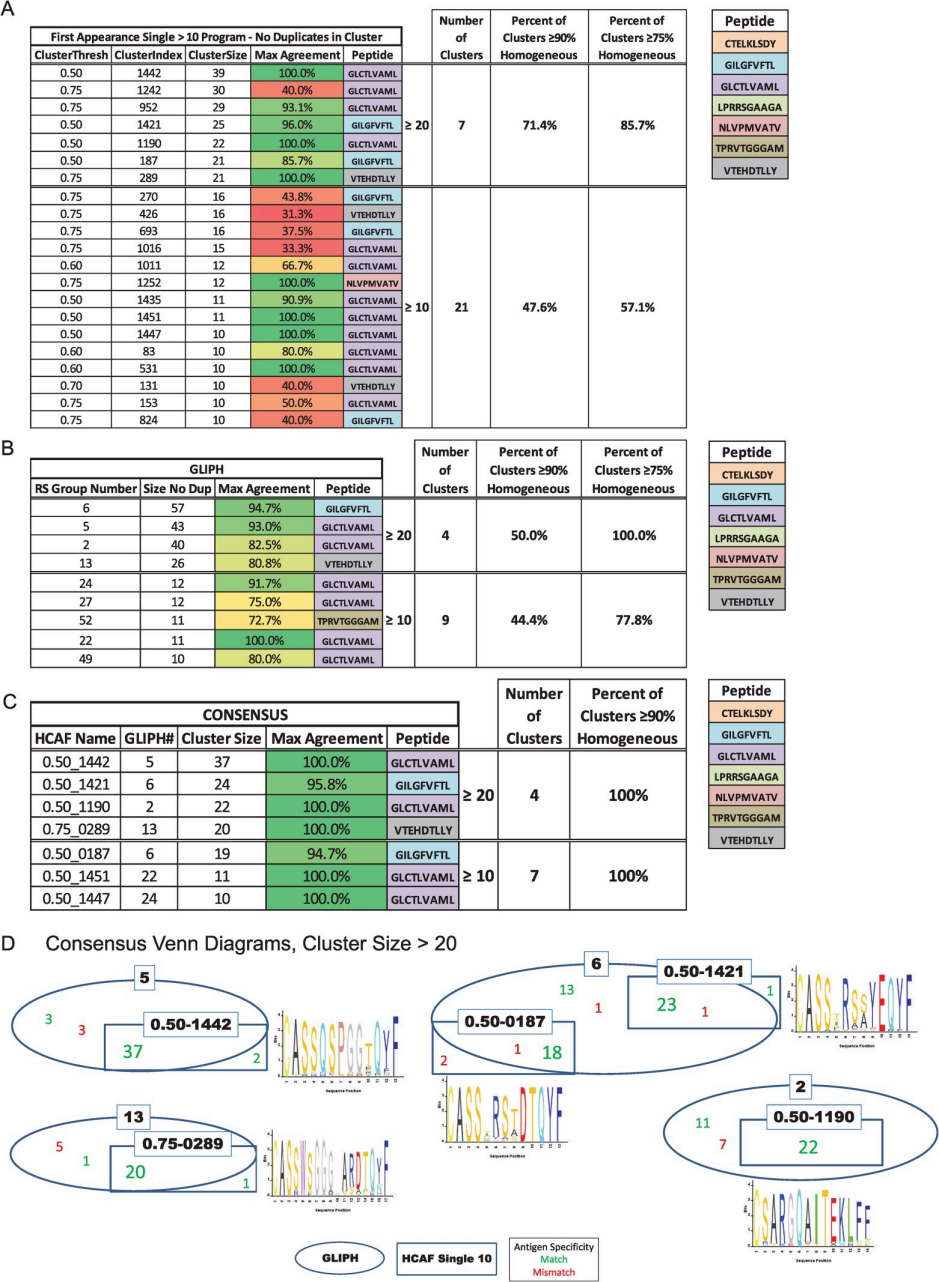
Comparison of GLIPH and HCAF methods to identify correct antigen specificity groups. TCRβ sequences of known antigen specificity were analyzed for peptide specificity using Hierarchical Clustering Appearing First (HCAF) (A) and GLIPH (B). **(A, B)** Clusters ≥ 10 or 20 unique sequences in size were assessed for their ability to group TCRs recognizing the same peptide. The data show the size of each cluster and the percentage of sequences in that cluster that bind the same peptide (max agreement). Homogeneity of the clusters for a single peptide is indicated by the percent of clusters that achieve > 75% or 90% homogeneity for the same peptide sequence. **(C)** Consensus clusters indicate clusters of TCR CDR3β sequences that were shared by antigen specificity groups generated by both GLIPH and HCAF. Tables show consensus cluster origin and size, and each consensus cluster was assessed as described above for the percentage of sequences in that cluster that recognize the same peptide (max agreement). This is also presented as the percent of consensus clusters that reach 75% and 90% homogeneity for a single peptide. (**D**) Representative diagrams of consensus clusters outlined in (C). Oval represents sequences found in GLIPH cluster and rectangle represents sequences found in the HCAF cluster. Where the oval and rectangle overlap is the consensus cluster. Green numbers indicate the number of sequences that match the correct peptide and red those sequences that bind a peptide other than the consensus. The consensus amino acid sequence of the Vβ region in each consensus cluster is provided.

Given the size and complexity of the predicted antigen specificity groups identified by GLIPH, we were interested in understanding if additional TCR clustering methods could identify similar antigen specificity groupings that likely share reactivity to the same or similar antigen. To do this we developed an algorithm based on TCR sequence similarity and cluster thresholding that we will be referring to in this paper as Hierarchical Clustering Appearing First (HCAF). HCAF is a variant of Agglomerative Hierarchical Clustering that differs from GLIPH in a few substantive ways. To validate the ability of HCAF to correctly identify antigen specificity groups, we used this approach to define clusters using a TCR reference set of known peptide-MHC specificity (Fig 4). HCAF and GLIPH clustering were applied to the same reference set. When assessing clusters possessing 90% homogeneity in their specificity for a single peptide, GLIPH and HCAF were able to accomplish this for only 44.4% and 47.6% of groupings containing 10 or more unique TCRβ sequences, respectively (Fig 4A and 4B). If the bar was lowered to 75% of TCR sequences in a cluster being specific for the given peptide, 77.8% of GLIPH clusters met this criterion compared to 57.1% of HCAF-derived clusters. When assessing clusters ≥ 20 unique TCRβ sequences, HCAF identified 71.4% of the clusters as being 90% homogeneous and 85.7% as 75% homogeneous for antigen specificity. In comparison, GLIPH remained at 50% when held to the 90% homogeneous criterion but identified 100% of clusters as being 75% homogeneous for a single antigen. Thus, both GLIPH and HCAF are better able to correctly identify the antigen specificity of groupings of 20 or more sequences than they are the specificity of smaller antigen specificity groups.

The overall ability to successfully group clusters into predicted antigen specificity groups using two distinct methods left open the question as to whether combining the two methods could further increase homogeneity of cluster reactivity toward a single antigen. To assess this, clusters generated by the intersection of GLIPH and HCAF clusters—i.e., those CDR3β sequences appearing in both the same GLIPH and HCAF clusters—were scored for their homogeneity to single antigens as described above. Using the combined HCAF-GLIPH consensus methodology, 100% of identified clusters met the 90% and 75% homogeneity criteria for a single antigen. This was true for clusters defined by ≥ 10 or 20 unique CDR3β sequences (Fig 4C). Representative consensus clusters identified in this manner and their consensus sequences are illustrated in Fig 4D. In summary, when GLIPH and HCAF are combined, those clusters that share a consensus of sequences identified by both methods are better predictors of correct antigen specificity than those determined using either method alone.

**Fig 5:**
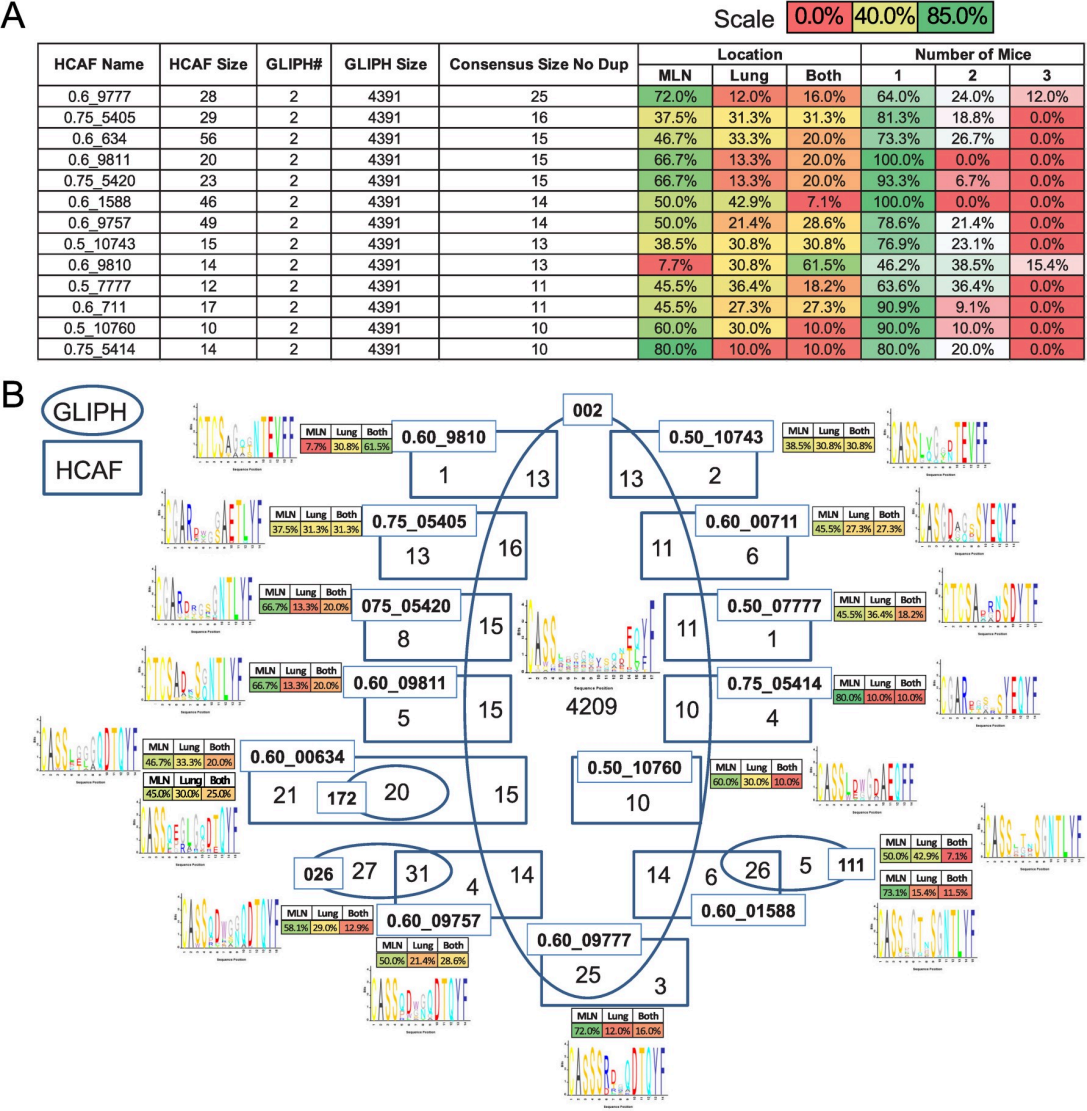
HCAF and GLIPH consensus clusters focus antigen specificity groups in the IL-4^+^ T cell repertoire. GLIPH and HCAF analysis was performed on bulk TCRβ sequences isolated from IL-4^+^ CD4^+^ T cells from the mediastinal lymph nodes and lung of IL-4^4get^ mice nine days after *N*. *brasiliensis* infection. **(A)** Table shows the size and distribution of consensus clusters shared between GLIPH and HCAF approaches within the largest identified GLIPH cluster. The relative proportion of sequences in each of the 13 consensus clusters is presented for their distribution across each tissue and mouse. **(B)** The diagram identifies HCAF consensus clusters within the largest GLIPH cluster and their consensus Vβ amino acid sequence.

We next applied the consensus HCAF-GLIPH approach to assess the bulk TCR repertoires obtained during helminth infection. We were particularly interested in the breakdown of the largest CRG group identified by GLIPH within the IL-4^+^ bulk CD4^+^ T cell repertoire. This CRG, denoted here as GLIPH #002, is synonymous with CRG group 1 in Fig 3A. It was composed of 4391 unique CDR3β sequences and could be broken down into 13 consensus groupings after pairing with HCAF clustering (Fig 5A and 5B). Although many of the sequences in these 13 clusters appear primarily in the mediastinal lymph node, the consensus clusters also contained sequences that were confined to only the lung (10.0–42.9%) or were found in both the lung and lymph node (10.0–61.5%) (Fig 5A). It is of interest to note that while many of the sequences in each consensus grouping appear specific to an individual mouse, all but one consensus group contained sequences that were found in 2 or more of the mice. The same approach was used to identify consensus clusters between GLIPH and HCAF analysis of the scTCRα/β dataset (S2 Fig).

### Transcriptome analysis reveals that IL-4-producing T cells in the lung and lymph nodes exhibit distinct gene signatures

**Fig 6:**
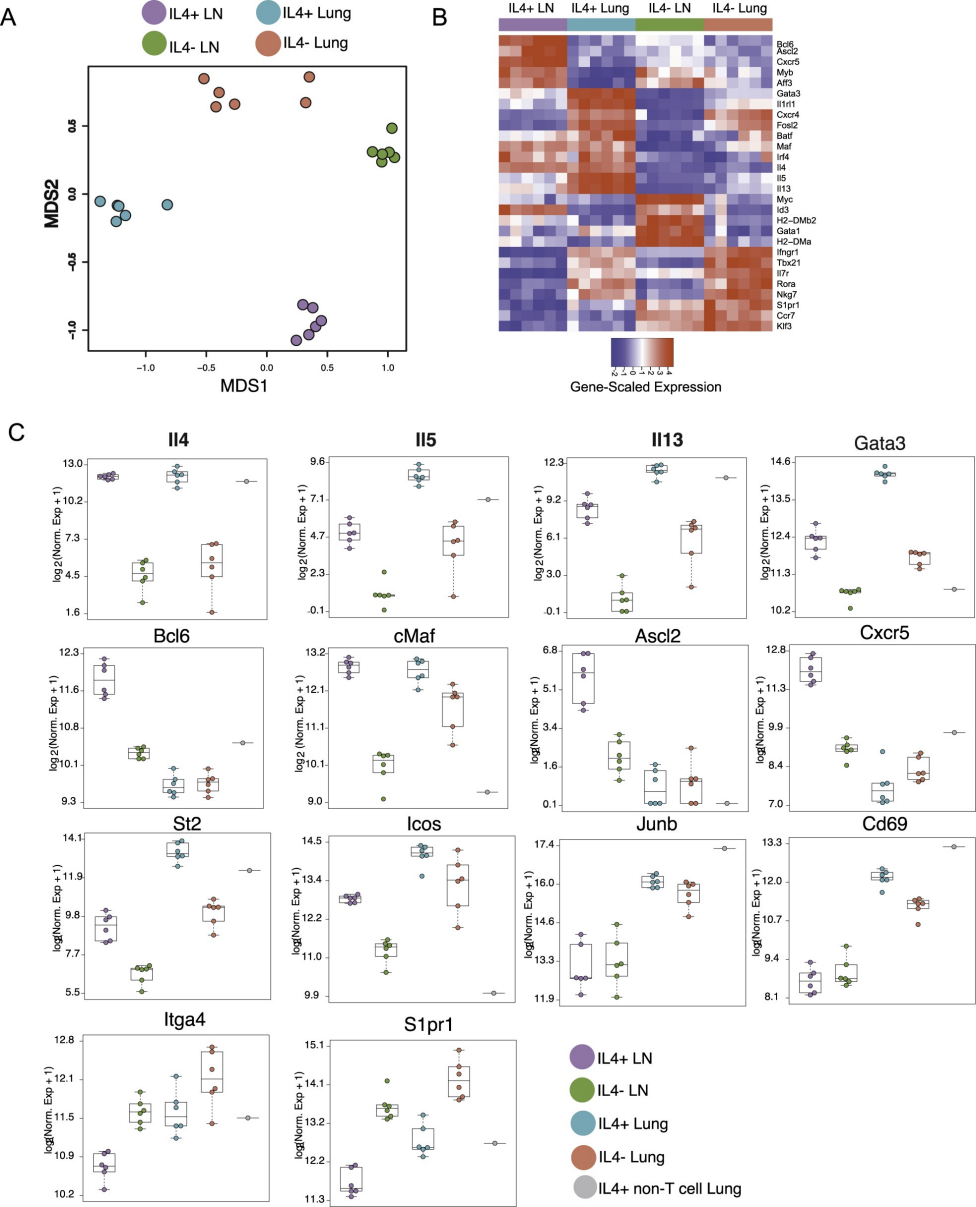
AmpliSeq reveals heterogeneity among IL-4-expressing CD4^+^ T cell populations in the lymph nodes and lung during helminth infection. IL-4^4get^ C57BL/6 mice were infected with *N*. *brasiliensis* and GFP^+^ and GFP^-^ CD4^+^ T cells from the lung and mediastinal lymph nodes were harvested and sorted nine days post-infection for bulk RNA AmpliSeq analysis. **(A)** Multi-dimensional scaling comparing IL-4-competent and IL-4^-^ CD4^+^ T cell populations isolated from the lung and lymph nodes of six mice infected on six different occasions. **(B)** Heat map showing the expression of select, characteristic genes in each CD4^+^ T cell population. **(C)** Log_2_-normalized expression of indicated genes within IL-4-competent and IL-4^-^ CD4^+^ T cell populations; n = 6, 1 mouse each from six different infections.

Shared TCRs with common antigen specificities among IL-4-competent CD4^+^ T cells in both the lung and lung-draining lymph nodes after helminth infection are intriguing when placed in the context of our understanding of Tfh and Th2 cell fate choice during *N*. *brasiliensis* infection. These subsets are often represented as distinct IL-4-producing cells in lymphoid and nonlymphoid tissues, respectively. Given that the TCR sequencing data show that the most prominent IL-4^+^ clones in the lymph nodes are also often found in IL-4^+^ lung populations and given lymph nodes are highly enriched for Tfh2 cells, while the lung is almost exclusively made up of Th2 cells at this time during infection, it is likely that many Tfh2 and Th2 cells share clonality. However, the data also reveal that some clonal selectivity is evident in these distinct tissues, suggesting that specific clones are also likely preferentially enriched among Tfh2 or Th2 cell phenotypes. To further investigate the relatedness between IL-4^+^ cells in nonlymphoid and lymphoid tissues, we compared the transcriptomes of GFP-positive and GFP-negative CD4^+^ T cells isolated and sorted from the lungs and mediastinal lymph nodes of six different IL-4^4get^ mice infected with *N*. *brasiliensis* (S3 Fig). Multidimensional scaling based on global gene expression analyses showed four distinguishable CD4^+^ T cell populations stratified by tissue and IL-4 expression (Fig 6A). Although the unbiased approach to investigate the anti-helminth response in this study did not allow the specific evaluation of transcriptomes from pre-sorted Tfh and Th2 cells, the bulk RNA-seq data clearly segregated IL-4^+^ CD4^+^ T cells in lymph node and lung into Tfh2-like and Th2-like cells, respectively, based on their unique gene signatures (Fig 6B and 6C).

**Fig 7:**
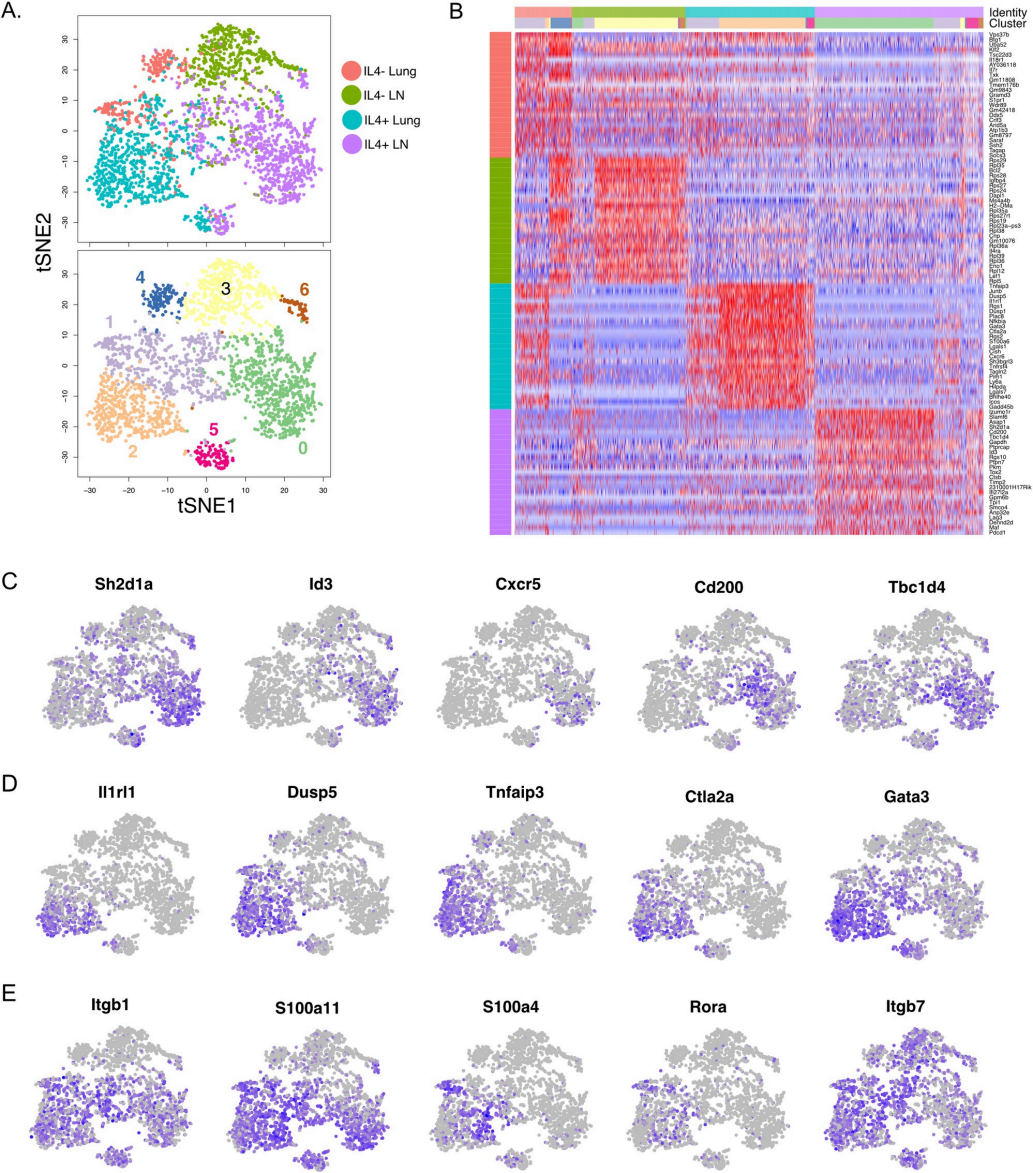
Single-cell sequencing reveals three distinct IL-4-expressing CD4^+^ T cell populations in the lymph nodes and lung. GFP^+^ and GFP^-^ CD4^+^ T cells from the lung and mediastinal lymph nodes harvested for one of the bulk RNA-sequencings was split and processed for single-cell RNA-seq analysis. **(A)** t-SNE analysis (Padj < 0.05) between GFP^+^ and GFP^-^ CD4^+^ T cells in the lung and mediastinal lymph node. Top plot: colors represent the four individual cell types isolated from the indicated tissues and each dot represents an individual cell. Bottom plot: unbiased k-nearest neighbor clustering was performed to identify six unique clusters. **(B)** Heatmap comparing the top 25 differentially expressed genes within each indicated CD4^+^ cell population with clusters designated by colors indicated in (A). **(C)** t-SNE analysis showing the relative expression of genes from cluster 0 (Tfh-like). (**D**) t-SNE analysis showing the relative expression of genes from cluster 2 (Th2-like). (**E**) t-SNE analysis showing the relative expression of genes from cluster 1 (shared between lymph node and lung; transitional).

To better assess the heterogeneity between lymph node- and lung-associated CD4+ T cell populations after helminth infection, single cell RNA sequencing (scRNA-seq) was performed to evaluate transcriptomes of individual cells. Supporting the bulk RNA-seq findings, IL-4-expressing (GFP^+^) and IL-4^-^ (GFP^-^) CD4^+^ T cells from the mediastinal lymph nodes and lungs fell into distinct populations that could be further stratified into 7 clusters based on their unique transcriptomes (Fig 7A). IL-4^-^ populations from the lymph node segregated into two clusters. While Cluster 3 remained largely independent of other CD4^+^ populations, Cluster 6 was shared between both IL-4^-^ and IL-4^+^ CD4^+^ T cell populations from the lymph node. This may reflect a population of cells that has yet to acquire IL-4 competency. Among non-IL-4-expressing CD4^+^ T cells in the lung, two distinct clusters are observed. Cluster 4 appears more similar to IL-4^-^ populations in the lymph nodes, while the second cluster makes up part of Cluster 1 sharing similarity with a subset of lung IL-4^+^ cells. This may reflect a circulating or transitory IL-4^-^ population that has yet to receive lung-specific signals required to achieve final commitment to a particular fate. Where tissue-resident memory populations fall along these clusters remains to be identified, but heterogeneity within the IL-4^-^ compartment in the lung is of interest to our understanding anti-helminth immunity.

Similar to IL-4^-^ populations, IL-4^+^ CD4^+^ T cells in the lung and lymph nodes exhibited significant heterogeneity. While IL-4-expressing CD4^+^ cells from the lymph nodes (purple) and lung (aqua) shared cells in clusters 1 and 5, clusters 0 and 2 were comprised almost exclusively of either lymph node or lung cells, respectively. Based on the differentially expressed genes in clusters 0 and 2, these cells represent Tfh and Th2 cells (Fig 7B). Cluster 0 is enriched for genes known to be involved in Tfh cell fate and function, including *Sh2d1a* (SAP1), *Id3* and *Cxcr5* (Fig 7C) [24–32]. Cluster 2, which is comprised of IL-4^+^ CD4^+^ T cells from the lung, is highly enriched for transcripts from genes known to be involved in Th2 fate maintenance including *Il1rl1*, *Gata3*, and *Tnfaip3* (Fig 7D) [33–38]. Cluster 1 represents a mixed population of IL-4^+^ CD4^+^ T cells from both the lymph node and lung. Of the differentially expressed genes assigned to this cluster, many reflect a migratory or transitory population destined for mucosal or inflamed tissues. This is highlighted by the integrin beta chains (Itgb) 1 and 7 (Fig 7B and 7E). *Rora* expression is also of interest here as this transcription factor does not appear to be required to mount normal Tfh or Th2 cell responses, suggesting that it may serve as a cell fate modulator similar to its role in innate lymphoid cell biology in settings of type-2 immunity [39,40]. Together, these findings confirm that tissue location is a key discriminator of IL-4^+^ T cell populations.

### Trajectory analysis reveals a potential ancestor between IL-4^+^ Tfh and Th2 cells

**Fig 8:**
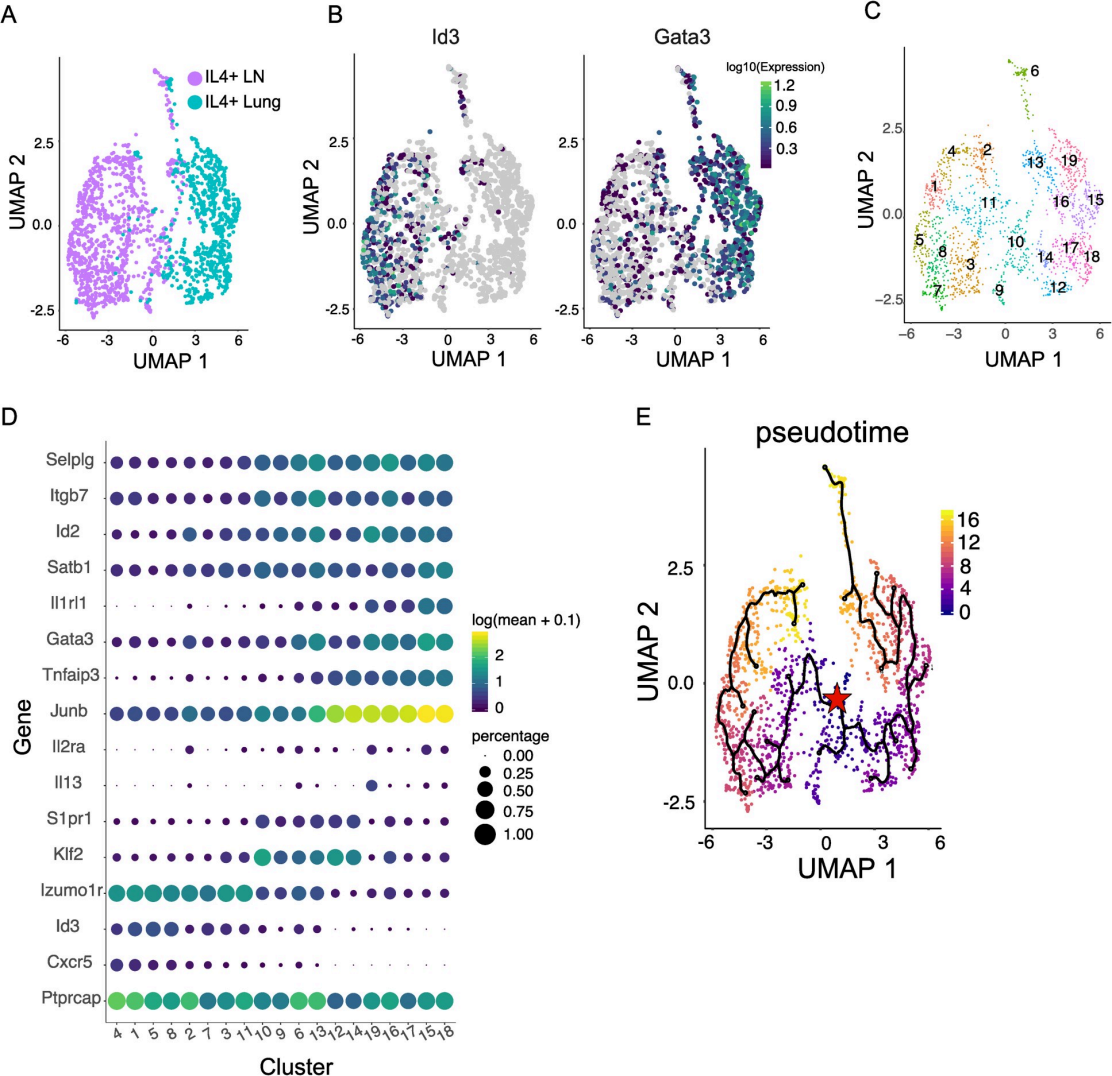
Trajectory analysis of IL-4^+^ T cells in the lymph node and lung after helminth infection. IL-4^+^ CD4^+^ T cells from the lung and mediastinal lymph nodes were harvested and processed for single-cell RNA-seq analysis. Trajectory analysis was performed using Monocle 3. **(A)** UMAP of IL-4^+^ CD4^+^ T cells in the mediastinal lymph node (purple) and lung (aqua). **(B)** UMAP overlaid with the relative gene expression of Id3 (left) and Gata3 (right) in single cells. **(C)** IL-4^+^ CD4^+^ T cells from the mediastinal lymph node and lung were clustered into 19 groups based on transcriptome relatedness. **(D)** Relative gene expression and the percentage of cells expressing indicated gene in each cluster as outlined in (C). **(E)** Pseudotime trajectory analysis starting at origin in cluster 10 (Red star). Pseudotime coloring represents an arbitrary period of time as cells progress along different branch points toward distinct cell fates.

Single cell transcriptomics showed that the majority of IL-4^+^ cells in the lung and lymph nodes exhibit distinct profiles, however, some cells from both of these tissues contained overlapping transcriptomes (Fig 8A). This is highlighted by purple and aqua cells sharing similar proximity in the Uniform Manifold Approximation and Projection (UMAP). If we overlay expression of the Tfh marker *Id3* and Th2 marker *Gata3* on these cells, those cells expressing the highest transcripts (lighter green) of *Id3* and *Gata3* fall on the respective peripheries of the lymph node and lung populations shown in the UMAP (Fig 8B). If expression from low to high is followed, the pattern is consistent with a more terminally differentiated state for both Tfh and Th2 cells at the periphery of the UMAP. However, if focused on where the lung and lymph node populations overlap (central region of UMAP), the cellular expression of these two genes is reduced relative to the expression of cells residing at the periphery of the UMAP. In some cases, we can observe centrally located cells expressing low levels of both *Id3* and *Gata3*. This pattern of increasing expression of genes characteristic of terminally differentiated Tfh or Th2 cells as cells fall further from the center of the UMAP plot is more clearly observed when comparing gene expression across 19 clusters that segregate distinct regions of the UMAP (Fig 8C). For example, if we start centrally at cluster 10 (which shares cells found in both the lung and lymph node) and follow the expression level of the Tfh-associated chemokine receptor *Cxcr5* to adjoining clusters to the left, there is a noticeable increase in expression in peripheral clusters such as 1, 4, and 5 (Fig 8D). A complementary pattern to what is observed for *Id3* expression. In contrast, if starting at cluster 10 and following the expression of the gene *Il1rl1* (encoding the IL-33 receptor and a marker of committed Th2 cells), expression increases as we move toward the periphery and encounter clusters 15, 16, 17, and 18 [41–43]. It should be noted that the genes known to regulate lymph node egress *S1pr1* and *Klf2* exhibit the highest expression in centrally located clusters 6, 9, 10, and 13 consistent with the transitory phenotype identified above. In support of their role in egress, these genes are decreased in both lymphoid-resident Tfh cells and nonlymphoid tissue-resident cells [44–46]. The overlap between the lymph node and lung cells at the center of the UMAP plot is interesting and suggests that cells obtained from both tissues are more similar to each other than their more committed Tfh and Th2 counterparts (Fig 8A and 8B). Whether this reflects a transitional population representing a shared precursor between committed IL-4^+^ Tfh and Th2 cells or circulating IL-4^+^ cells found in each tissue that have either lost their tissue-specific signature or have yet to acquire it is not clear.

In an effort to better identify the nature and relationship between IL-4^+^ T cells in the lymph nodes and lung with the putative transitional population found in both tissues, we performed trajectory analysis designed to predict how cells might progress between or toward distinct tissue fates using a single snapshot of time in gene expression. To do this we employed Monocle 3 (http://cole-trapnell-lab.github.io/projects/monocle/) [47,48]. The Monocle 3 toolkit allows for the construction of single cell trajectories from single-cell RNA-seq datasets and builds a learned trajectory for each cell. Each branch in the learned trajectory represents a different cellular decision. By assessing where the transitional population of cells fall along the trajectory and its branches, insight can be gained in the relatedness between this population and more committed Tfh and Th2 populations. When a trajectory run begins at this intersection of similarity between lung and lymph node T cells (red star, Fig 8E) two clear branch points are observed moving either toward a more committed Tfh (CXCR5^high^, Id3^high^) or Th2 (Il1rl1^high^, GATA3^high^) fate. Consistent with the above analysis of more terminal gene expression residing in the periphery of the UMAP, the trajectory in both cases moves outward from this “transitional” point of origin. This finding is consistent with a transitional population of IL-4-expressing T cells that can acquire either Tfh or Th2 cell fate.

### Unique chromatin landscapes are enriched in IL-4-expressing T cells residing in different tissues

**Fig 9:**
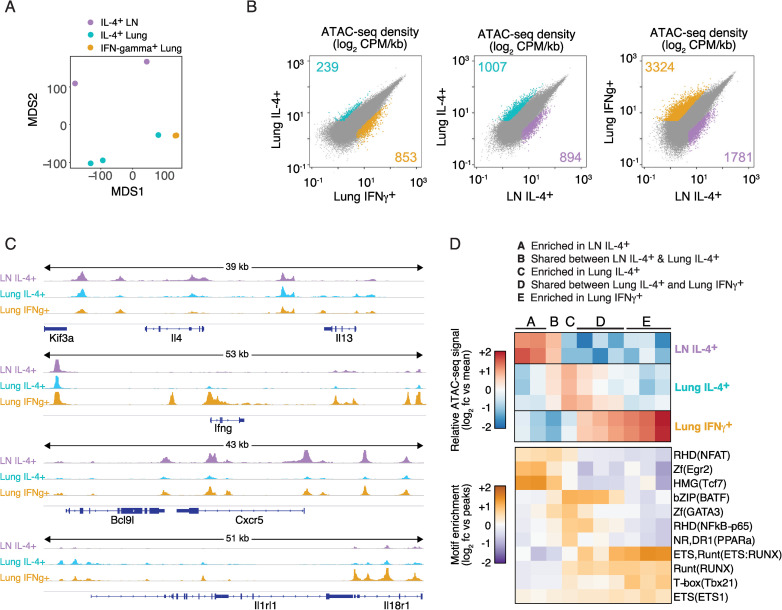
ATAC-sequencing reveals distinct chromatin landscapes among IL-4-expressing CD4^+^ T cells in the lymph nodes and lung. IL-4^4get^ and IFN-gamma^GREAT^ C57BL/6 mice were infected with *N*. *brasiliensis* and GFP^+^, YFP^+^, and reporter-negative CD4^+^ T cells from the lung or mediastinal lymph nodes were harvested and sorted eight days post-infection for ATAC-sequencing. **(A)** Multidimensional plot using all accessible chromatin regions to assign similarity among indicated CD4^+^ T cell subsets. **(B)** Diagonal scatterplots of mean ATAC-seq counts per peak comparing accessible chromatin regions in the indicated populations. Each point represents a single peak from the ~86,000 peaks observed in the three populations. Colors designate if a peak is differentially accessible (>3-fold change) between the two populations (Padj < 0.05). **(C)** Mean ATAC-seq coverage for *Il4*, *Il13*, *Ifng*, *Cxcr5*, and *Il1rl1* loci with a scale of 0–400 for all tracks. **(D)** Top: k-means clustered heatmap of log_2_ fold-change from global mean at all peaks among indicated IL-4- and IFN-gamma-expressing CD4^+^ T cell populations. Bottom: relative motif enrichment among unique peaks from each cluster (Top) are compared to all ATAC-seq peaks; n = 2–3 mice, from a single experiment.

Gene expression patterns among cells involved in type-2 immunity may be controlled by specific sets of regulatory elements that influence cell fate and function [2,49–51]. To identify shared and unique regulatory elements within the chromatin landscape of IL-4-expressing CD4^+^ T cells in the lymph nodes and lung, we used Assay for Transposase-Accessible Chromatin using sequencing (ATAC-seq). Specifically, ATAC-seq identified regulatory regions unique to GFP^+^ and GFP^-^ CD4^+^ T cells isolated from the lung and mediastinal lymph nodes of IL-4^4get^ mice infected eight days prior with *N*. *brasiliensis*. As an added control for type-2 specificity at this time point, we also included IFN-gamma expressing CD4^+^ T cells from the lung of *N*. *brasiliensis*-infected IFN-gamma^GREAT^ reporter mice [52]. From these analyses, we identified 85,834 regions that were accessible in at least one cell type. To compare between cell types, we computed the number of transposase insertions within these regions (ATAC-seq density) for each replicate. When comparing ATAC-seq signal between all replicates across all regions we found that IL-4-expressing CD4^+^ T cells from the lung clustered independently from the IL-4-expressing CD4^+^ T cells isolated from the lymph nodes (Fig 9A). Of particular note, the IL-4-expessing CD4^+^ T cells from the lung of one mouse showed striking similarity to IFN-gamma (IFNγ)-expressing CD4^+^ T cells from the lung of two different mice. This suggests that these Th2 cells may share similar regions of chromatin accessibility as more conventional Th1 cells. In identifying regions that were differentially accessible between each sample type, we found 239 peaks with higher signal in IL-4-expressing CD4^+^ T cells in the lung compared to lung-associated IFNγ-expressing CD4^+^ T cells, while 1007 unique peaks were observed when this same population was compared to IL-4^+^ CD4^+^ T cells in the lymph nodes (Fig 9B). Examples of unique peaks at representative loci associated with Th1, Th2, and Tfh cells are shown in Fig 9C. Together, the increased similarity in accessible chromatin between IFNγ^+^ and IL-4^+^ CD4^+^ T cells in the lung as compared to the similarity of accessibility peaks shared between IL-4^+^ cells in the lung and lymph nodes suggests that tissue residency is an important discriminator of cell identity. Given that regions of open chromatin are associated with gene expression, it is also likely that tissue-specific changes in chromatin accessibility contribute to the tissue-specific transcriptomes observed among IL-4^+^ CD4^+^ T cells in the lung compared to their IL-4+ counterparts in the lymph nodes. Following this logic, tissue-specific changes to the chromatin landscape are likely also playing a key role in differential gene expression observed in lymph node-residing Tfh cells and lung-residing Th2 cells. This again highlights the importance of tissue residence in the regulation of distinct gene sets.

To identify regulatory elements with shared or unique profiles between tissues and cell subsets we partitioned peaks that were differentially accessible in at least one of the three populations described above (lymph node IL-4^+^, lung IL-4^+^, and lung IFNγ^+^) using k-means clustering and assigned clusters with similar motif enrichments into 5 groups designated A, B, C, D, and E (Fig 9D). To identify potential transcription factors that may be associated with these regulatory elements, the enrichment of known transcription factor motifs in each group compared to all peaks was determined (Fig 9D). Although many motifs were enriched in peaks shared across all or two of the cell types (clusters B, D), unique enrichment of motifs in each population was also observed (clusters A, C, E). Expectedly, runt-related transcription factor (Runx) and T-box (T-bet) motifs were enriched among the accessible regions unique to IFN-gamma-expressing CD4^+^ T cells in the lung (cluster E) [53–57]. Similarly, GATA, PPAR, and bZIP transcription factor motifs were enriched in IL-4^+^ cells from the lung (cluster C) consistent with the RNA-seq data presented here and previous literature on Th2 cells [34,35,58–62]. Cluster A, consisting of accessible regions unique to IL-4-expressing CD4^+^ T cells in the lymph node, was enriched for the Rel homology domain (RHD), nuclear factor of activated T cells (NFAT), zinc finger transcription factor early growth response 2 (EGR2), and high mobility group (HMG) T-cell factor (TcF) motifs. These enriched motifs represent transcription factors important for Tfh cell function and generation [63–67]. For the accessibility peaks shared by all IL-4^+^ CD4^+^ T cells regardless of tissue residence (cluster B), the motif enrichment demonstrated a mixture of factors including bZIP and GATA. This is consistent with cluster B identifying accessible peaks that are enriched for transcription factors important in both Tfh and Th2 cell function. Together, the ability to identify transcription factor family motifs that are enriched among accessible regions unique to Tfh and Th2 cells helps to inform how certain transcription factors may be regulating cell commitment and function in these distinct subsets.

## Discussion

The nature of the host response to *N*. *brasiliensis* is complex. The data herein revealed that the responding pool of IL-4^+^ CD4^+^ T cells in the lung and lymph nodes after helminth infection represented distinct populations based on their unique gene expression and chromatin accessibility profiles, but shared a common TCR repertoire and overall antigen reactivity. Within a given animal, clones and clonotypes were often shared between the mediastinal lymph node and the lung. Although the most abundant IL-4^+^ clones and clonotypes were found in both the lymph node and lung compartments, the top clones in each of these groups exhibited some tissue selectivity or enrichment. Across different animals, repertoires were distinct and few public clonotypes observed. However, the antigen specificity of these unique repertoires was predicted to be shared across both tissues and mice. Together the data suggest that, while overall the antigen reactivity of the CD4^+^ T cell response is common between different mice exhibiting distinct IL-4^+^ repertories, there is some preferential clonal enrichment observed within these two tissue compartments.

The overlapping nature of IL-4^+^ clonotypes and clones in lymphoid and nonlymphoid tissues—despite the unique, tissue-specific nature of their transcriptomes and chromatic accessibility—is interesting in the context of what is currently understood with respect to Tfh and T helper fate choice. Tfh and Th cell fate choice is dependent on both environmental access to IL-2 as well as the affinity of the TCR and the strength and duration of signal achieved during differentiation [68–74]. Although there is still much debate as to whether strong or weak signals selectively promote fate choice between Tfh and Th1 subsets [69,70,74–76], responsiveness of the TCR helps to segregate cells into either lymphoid-restricted Tfh or nonlymphoid Th compartments. Selection toward a Th or Tfh fate may also include differences in tonic signaling [77]. In its simplest form, the idea of a preferential fate choice based on TCR signal strength or affinity for antigen implies that Tfh and non-Tfh subsets—and by association their respective tissues of residence—likely possess different TCR repertoires. Indeed, the preferential recruitment of specific TCRs into Tfh and non-Tfh subsets bares out in many experimental systems that rigorously track antigen-specific T cells using peptide-MHC-tetramers and TCR-transgenic cells [69,70,75,77]. Evidence of selective repertoires also exists when more polyclonal responses with broader antigen specificity are investigated [78]. However, selectivity of repertoires among Tfh and non-Tfh cells may not be the case in all circumstances. This is particularly true in humans where assessment of the repertoires among circulating Tfh cells, lymphoid-resident Tfh cells, and lymphoid-resident, non-Tfh cells show substantial clonal overlap [79,80]. Nevertheless, it should be noted that such findings may be complicated by non-Tfh populations containing Tfh-like cells [81]. Biologically, the reality between shared and selective Th and Tfh repertories is likely somewhere in between. This is evident both from the study presented herein and from a recent study of the CD4^+^ T cell responses to lymphocytic choriomeningitis virus (LCMV) infection [82]. Although the majority of naïve clones appeared to give rise to multiple cell subsets in this study, approximately a quarter of the clones exhibited some selectivity for either Tfh or non-Tfh subsets. Here we find similar results in response to *a* helminth infection. When focusing on the most abundant clones and clonotypes generated in response to *N*. *brasiliensis* infection, many were found in both the mediastinal lymph node and lung of the same animal. Given that most of the IL-4-expressing cells in the lymph nodes at this timepoint are Tfh2 cells and those in the lung are Th2 cells, it follows that many Tfh and Th2 cells share the same TCR. Thus, while on an individual basis the strength or quality of signal mediated through the TCR may predispose a naïve cell toward a given Tfh or Th2 fate, when the polyclonal repertoire is assessed, the majority of clones appear to have the capacity to populate both subsets. From an immune perspective, this shared clonality is conceptually important as it links the antigen reactivity of the humoral response in the lymph node (i.e., that driven by Tfh cells) to the cell-mediated response (i.e., that driven by Th2 cells) in the lung. This ensures that these distinct but synergistic arms of the adaptive immune response can work in concert to promote effective immunity to helminth infection. How clonal restriction in memory responses changes the constituency of these subset repertoires will be an important next step in our understanding of immunity to helminths.

When extending our analysis to assess if antigen specificity of the polyclonal, IL-4^+^ CD4^+^ T cell pool is maintained across different mice, three things become clear. First, antigen specificity groups are rarely dominated by a single clone or clonotype. Second, antigen reactivity of the CD4^+^ T cell repertoire is conserved across mice and tissues not through the generation of public clones or clonotypes but through CD4^+^ T cells expressing distinct TCRs that are predicted to bind similar antigenic peptides. Third, while certain clones may preferentially exist in the lymph node and lung, antigen specificity groups are made up of several clones which span both tissues. Together this implies that, despite some selectivity of a given TCR for maintenance in a particular tissue (i.e. lymph node or lung), it is the breadth of the CD4^+^ T cell response in the context of helminth infection that largely increases the likelihood that antigen reactivity is shared in both tissues. As discussed above, the equivalent antigen reactivity predicted in the lymph nodes and lung helps to pair the humoral response to the appropriate cell-mediated response against *N*. *brasiliensis*. The shared antigen reactivity among the IL-4^+^ T cell repertoires in the lymph node and lung is also intriguing with regard to the putative transitional IL-4^+^ CD4^+^ T cell population. While definitive experiments pairing TCR and transcriptome analysis have yet to be done to fully understand the relationship of this intermediate population to more committed Tfh2 and Th2 populations, data presented here are most consistent with this population sharing some clonality and antigen specificity with both subsets. If this represents a circulating population that transits between the lymphoid and nonlymphoid tissues throughout infection, it is likely to further cement a link between antigen responses in the lymph nodes and those at the site of infection. Specific clonal assessment of this population during primary and secondary infection will be important for our understanding of how this population contributes to Tfh2 and Th2 cell repertoires over time.

A number of algorithms have been developed to address the antigen specificity of responding CD4^+^ T cell repertoires. These have been validated against known and dominant epitopes residing in *Mycobacterium tuberculosis*, influenza virus, cytomegalovirus, and Epstein-Barr virus [22,23,83]. However, no such study has been conducted on repertoires generated in response to antigenic loads similar to those found in *N*. *brasiliensis* infection. To put this in context, the *M*. *tuberculosis* genome is 4.4 Mb and predicted to have 4000 coding genes [84–86]. The *N*. *brasiliensis* genome is predicted to be between 255–270 and 294 Mb with over 22,796 coding genes according to different genome assemblies [87]. The antigenic load also changes during the course of *N*. *brasiliensis* infection further increasing the complexity of the antigens available during infection. This is nicely shown as changes in the proteome as *N*. *brasiliensis* develops from the infectious larval stage to the adult colonization stage in the host [7,8]. Consistent with the numerous antigens available, the GLIPH algorithm used to assign T cells into groups of similar antigen specificity revealed a diverse repertoire with broad antigen reactivity [22]. While many of these groupings did show clear similarities across their TCRs, it was not possible to define a consensus TCR sequence in larger antigen specificity groups with this method. However, when GLIPH analysis was combined with novel HCAF clustering, 13 consensus clusters sharing TCRs between both methods were identified, and these clusters exhibited distinct but related amino acid sequences. Our data shows that the ability to breakdown large clusters into smaller HCAF-GLIPH consensus groups is likely more predictive of shared antigen specificity as the HCAF-GLIPH consensus method was significantly better than either method alone at grouping sequences of the same antigen reactivity. In fact, 100% of clusters sized greater than 10 unique clones were at least 90% homogeneous for a single peptide specificity as determined by a known reference repertoire. Thus, for large repertoires, responding to diverse and unknown antigens, HCAF-GLIPH consensus groupings are expected to aid in identifying TCRs of a particular antigen specificity.

Given the breadth of the anti-helminth response, identification of dominant or common antigens relevant to the protective response to *N*. *brasiliensis* has proved difficult. Similarly, whether the IL-4^+^ CD4^+^ T cell repertoire residing in the lung and mediastinal lymph nodes are representative of repertoires found in other tissues remains unclear. For example, it is likely that distinct repertoires exist in the lung compared to the intestine. The rationale for this is the developmental stage- and tissue-specific nature of *N*. *brasiliensis* as it progresses through its lifecycle. The infectious L3 larvae of *N*. *brasiliensis* first infect through the skin, then enter the bloodstream and arrive in the lung via the capillary beds. Once in the lung, the L3 larvae transition to the L4 stage as they migrate into the airways and trachea prior to colonization of the small intestine, where worms mature into L5 adults. As each stage of the helminth life cycle is associated with a unique proteome, each stage and tissue is likely to promote a distinct CD4^+^ T cell response [7]. As a result, the distinct proteomes associated with these different tissues are likely to have implications for vaccine design and epitope discovery. Vaccines against human hookworms that target both larval and adult antigens have shown promise. However, each approach has been met with some difficulties. Vaccines targeting the infectious larval stage of *Necator americanus*, while effective, cause urticaria in upwards of 40% of individuals that live in areas endemic to hookworm [88,89]. Vaccines targeting proteins involved in feeding of the adult worm have also shown promise, but these do not prevent infection or limit the tissue pathology in the skin and lung associated with early stages of infection [90–92]. Whether such issues might be overcome through further exploration of antigenic targets or the development of relevant mimotopes is something to be explored. The repertoire analysis provided here in relation to responses to *N*. *brasiliensis* provide a framework in which to identify such antigens.

Despite the high degree of clonality and sharing of predicted antigen specificity between IL-4^+^ T cells in the lymph nodes and lung, the transcriptomic data showed that IL-4-competent CD4^+^ T cells in the lung and lung-draining lymphoid tissues can be separated into distinct populations. The unique tissue identity imparted on these IL-4^+^ cells in the lymph nodes and lung moves counter to the classical and oft-cited paradigm that Th2 cells are responsible for both humoral and cell-mediated hallmarks of type-2 immunity [10,93–95]. Instead, when these findings are placed in the context of Tfh cell biology, they support a more bifurcated view of how type-2 cytokine-expressing CD4^+^ T cells orchestrate immune responses to type-2 pathogens. This bifurcated model of type-2 immunity posits that canonical Th2 cells modulate peripheral immunity through the production of IL-4, IL-5, and IL-13, while IL-4-producing Tfh cells reside primarily in the B cell follicles to promote B cell-driven IgE and IgG1 production. A parallel bifurcation between humoral and cell-mediated immunity in peripheral tissues can also be gleaned from CD4^+^ T cell responses to viral or bacterial infection. In type-1 settings, Tfh cells help B cells while Th1 cells activate macrophages at peripheral sites of infection [69]. This division of cellular labor among responding Tfh and T-helper cells provides the coordination necessary to pair the appropriate antibody response with cell-mediated immunity to best control the invading pathogen. Datasets obtained here pairing ATAC-seq with bulk- and scRNA-seq provide a foundation to reveal transcription factors and related gene sets that are important in the shared or selective function of Tfh or Th2 fates. The tissue-specific pathways important in driving this functional bifurcation will be important to our understanding of anti-helminth immunity and type-2 inflammation in general.

Where cytokine competency and fate commitment occur among IL-4-competent CD4^+^ T cells has been an area of recent investigation. Tfh fate specification and commitment is believed to occur early in response to antigen [96,97], with maintenance and commitment of Tfh fate requiring entry into the B cell follicles and persistent antigen [98,99]. Once in the B cell follicles, Tfh cells are believed to acquire IL-4 expression [100,101]. Similarly, terminal Th2 cell commitment occurs only after IL-4-competent cells exit the lymph nodes and become licensed by alarmins in the lung [2]. The data presented here suggests that such models may be too restrictive. It is interesting to speculate as to the origin and nature of IL-4-expressing cells whose transcriptomes and chromatin landscapes segregate away from both canonical Th2 (GATA3^+^) cells in the lung and Tfh (Id3^+^CXCR5^+^BCL6^+^) cells in the lymph nodes. The trajectory analysis performed herein is consistent with the possibility that an IL-4^+^ precursor can give rise to both IL-4^+^ Tfh and Th2 cells. This was supported by the increasing expression of key Tfh- and Th2-related gene sets in cells as branching or fate-decisions moved further away from this putative transitional cell of origin. The IL-4^+^ transitional population described herein is consistent with a recent study showing that gene programs and chromatin landscapes associated with IFN-gamma and IL-17-expressing expressing CD4^+^ T cells in the intestine were not easily segregated into classical Th subsets but instead rested along a continuum dictated in large part by the nature and timing of the infection [102]. In sum, the data provided lay the foundation to determine when IL-4 expression is acquired, the fate and plasticity of this transitional population, and the role this transitory IL-4^+^ CD4^+^ T cells plays in host defense and immunologic memory to helminths.

In summary, the combining of cytokine reporter mice with the novel HCAF-GLIPH consensus method described herein and other single cell approaches provide opportunities to explore T cell responses to complex pathogens or antigens in an unbiased manner and help to clarify new paradigms related to the clonal relationship, transcription factors key in lineage-specification, and fate commitment of Tfh and Th2 cells in the context of helminth infection. In addition, the data provides new insight into the tissue-specific nature of type-2 immunity which contributes significantly to both the heterogeneity as well as the regulation of T cell responses in lymphoid and nonlymphoid tissues in the context of complex pathogens.

## Materials and methods

### Ethics statement

All studies were performed in accordance with the principles described by the Animal Welfare Act and the National Institutes of Health guidelines for the care and use of laboratory animals in biomedical research. All procedures were approved by the Institutional Animal Care & Use Committee (IACUC); the Division of Laboratory Animal Resources at Duke University Medical Center; and the Biological Resource Center at National Jewish Health.

### Mice

IL-4^4get^, IL-4^KN2^, and IFNγ^GREAT^ mice were kindly provided by Richard Locksley (UCSF) and have been previously described [19,21,52]. Mice were bred and maintained on reporter C57BL/6 and BALB/c genetic backgrounds at Duke and National Jewish Health. Mice were maintained in specific pathogen-free animal facilities.

### Infections

*N*. *brasiliensis* was prepared as previously described [103]. Mice were infected with 500 L3 larvae in 200 uL PBS subcutaneously in the lower back.

### Tissue digestion for flow cytometry and sorting

Lungs were chopped with a razor blade, digested with 250 μg/ml Collagenase XI (C7657; Sigma), 50 μg/ml Liberase (145495; Roche), 1 mg/ml Hyaluronidase (h3506; Sigma), and 200 μg/ml DNase I (DN25; Sigma) in RPMI 1640 at 37°C for 30 minutes. Tissues were pipetted vigorously every 15 minutes until well digested. Tissue fragments were then filtered through an 80-micron mesh, washed, lysed for 2 minutes with ACK buffer to reduce the presence red blood cells, washed with PBS, and resuspended in 2% FBS in PBS. Single cell suspensions of lymph nodes were prepared by mechanical dissociation. All tissues were filtered immediately prior to sorting and data collection.

### Staining for flow cytometry and sorting

Fc receptors were blocked (1:100; Trustain FcX Biolegend) for 15 minutes prior to antibody staining. All stains were performed at 4°C for 30 minutes unless otherwise stated, using the following antibodies in 2% FBS in PBS: anti-mouse CD3ε (145-2C11); anti-mouse CD4 (RM4-5); anti-mouse CD8α (53–6.7); anti-mouse CD11c (N418); anti-mouse CD11b (M1/70); anti-mouse CD45 (30-F11); anti-mouse/human CD45R/B220 (RA3-6B2); anti-mouse CD279/PD-1 (RMP1-30); anti-mouse NK1.1 (PK136); anti-mouse Ter119 (Ter 119); and streptavidin were purchased from Biolegend. Anti-human CD2 (S5.5) was purchased from Invitrogen. Anti-rat/mouse CD185/CXCR5 (2G8) were purchased from BD biosciences. Prior to analysis, cells were resuspended in 2% FCS in PBS containing DAPI (0.5 μg/ml) to discriminate live cells. Lymphocyte and singlet gates were performed by size and granularity based on forward and side scatter. Cells were sorted using a FACSAria (BD Biosciences) and data was collected on a Fortessa or LSR II (BD Biosciences) cytometer and analyzed using FlowJo (TreeStar).

### TCRβ and paired TCRα/β sequencing

**Littermates of** IL-4^4get^ C57BL/6 (male) and BALB/c (female) mice were infected with *N*. *brasiliensis*. Mice were perfused with PBS and the lung and mediastinal lymph nodes were harvested at the indicated days post-infection. Lungs were enzymatically digested as described above and single cell suspensions were prepared. For TCRβ sequencing, 60,000–100,000 IL-4-competent CD4^+^ T cells (DAPI-negative, CD45^+^, CD4^+^, CD11b^-^, CD8^-^, B220^-^, GFP^+^) were sorted from each individual tissue into separate 1.5 mL Eppendorf tubes containing PBS (w/ 2% FBS and 0.025M HEPES buffer). Cells were spun at 986 rcf for 4 minutes and the pellet was washed twice. Cell pellets were dried and frozen at -80°C. Frozen pellets were processed for genomic DNA as recommended by Adaptive Biotechnologies mouse TCRβ immunosequencing kit. Amplification, library preparation, and sequencing of TCRβ CDR3 was performed using the immunoSEQ Platform (Adaptive Biotechnologies, Seattle, WA). The immunoSEQ Platform combines multiplex PCR with high-throughput sequencing and a sophisticated bioinformatics pipeline for TCRβ CDR3 analysis [104,105]. Jaccard index analysis was performed using ImmunoSEQ Analyzer 3.0 Software (Adaptive Biotechnologies). For scatter plot analysis, only sequences present at least twice within the samples were analyzed. The raw counts (at the nucleotide or amino-acid level) for each sample were log-normalized and the resulting values for each sample were plotted against every other sample to identify those with the highest degree of TCRβ sequence sharing.

For paired TCRα/β sequencing, at least 150,000 CD4^+^ T cells were sorted of each group and cells were submitted at a concentration of 1000 cells/ul for library preparation using Chromium Next GEM Single Cell 5’ Library and Gel Bead Kit v1.1 (10x Genomics) using the Chromium Controller according to the manufacturer’s protocol. Prior to library construction, the mouse T cell Chromium Single Cell V(D)J Enrichment Kit (10x Genomics) was used to enrich 10x barcoded, full length VDJ regions using primers specific to the mouse TCR constant region. Single cell libraries were sequenced on an Illumina NovaSeq 6000. V- and J-family assignments, identification of CDR3 sequences and calculation of clonotype frequencies were performed using Cell Ranger 3.0.2 (10x Genomics) with 10x VDJ reference data version 2.2.0, which is based on GRCm38 mouse genome assembly and Ensembl gene annotations. Only paired TCRs with a single alpha and beta chain are included in the analysis. Treemaps were generated to visualize differences in clonal diversity using the treemap package (v2.4–2; https://CRAN.R-project.org/package=treemap) in R (v3.5.1). Jaccard index similarity between samples was computed using the jaccard R package (v0.1.0; https://CRAN.R-project.org/package=jaccard).

### Prediction of antigen specificity groups

A table of TCR beta sequences (columns CDR3b, TRBV, TRBJ, and PATIENT) from each mouse and tissue was compiled and analyzed in GLIPH [22]. The PATIENT column was designated as the tissue and mouse from which TCRβ sequence was derived. An optional reference database (option—refdb) was used comprising 107,990 unique TCRβ sequences from the spleen of two unselected, naïve C57BL/6 mice (Adaptive Biotechnologies control set). Balb/c repertoires were compared to a naïve Balb/c reference data set (Adaptive Biotechnologies control set). Default parameter of 1000 random samplings against the naïve repertoire was used for both local and global TCR convergence. Convergence or similarity groups were clustered based on TCRs that share global similarity (i.e., differ by ≤ 2 aa) or share a significant motif (>10-fold enriched above naïve reference pool and <0.001 probability of being enriched to a similar extent in the naïve reference pool). PATIENT (unique tissue and mouse identifier) column was applied to the outputted clone network table and used to assign nodes and edges that were similar between tissues and mice. The network was mapped using Cytoscape (v.3.7.1) where nodes (clones) were colored either by which tissue (Fig 3B) or by how many mice (Fig 3C) they appeared within.

HCAF clusters were determined using the following approach. Sequence distances were determined using the Juke-Cantos method[106], where if *p* is the proportion of mismatched amino acids then the distance $d=-\frac{19}{20}\ln\left(1-\frac{20}{19}p\right)$. Agglomerative Hierarchical Clustering was then performed on these distances using single linkages [107]. Inconsistency coefficients (standardized deviations from the mean cluster height at each linkage) were calculated for each linkage of the cluster, and clusters were identified based upon these values. In particular, when ranging over inconsistency coefficients from 0.50 to 1.10, at the first appearance of a given agglomeration of size 10 or more was formed, that cluster was sequestered and larger agglomerations containing it were not considered. In this manner the algorithm uses the benefit of single linkage so that closely-related sequences will group together, while minimizing the detrimental effects of chaining.

### Targeted transcriptome RNA sequencing

IL-4^4get^ C57BL/6 mice were infected with *N*. *brasiliensis* and the lung and mediastinal lymph nodes were harvested nine days post-infection. Single cell suspensions of the lungs and mediastinal lymph nodes were prepared. IL-4^+^ CD4^+^ T cells (DAPI-negative, CD3ε^+^, CD45^+^, CD11b^-^, CD11c^-^, CD8^-^, B220^-^, NK1.1^-^, Ter119^-^, GFP^+^) and IL-4^-^ (GFP^-^) CD4^+^ T cells were sorted at 4°C. Sorted suspensions were centrifuged and resuspended in 300 μL of 2% FCS in PBS and counted to verify flow numbers before RNA extraction and single cell sequencing. For targeted transcriptome RNA-seq, sample RNA was isolated using the Quick-RNA Microprep kit (Zymo Research) according to the manufacturer’s protocol. Bulk RNA-seq gene expression data was generated using the Ion AmpliSeq Transcriptome Mouse Gene Expression Kit (ThermoFisher Scientific) using 2 nanograms of total RNA according the manufacturer’s protocol. Briefly, the sequencing library generation protocol involved the following steps: cDNA generation, amplification of amplicons for >20,000 gene targets using the AmpliSeq Mouse Transcriptome primer panel, and ligation of sequencing library adapters. Quality and quantity of libraries were assessed using an Agilent Bioanalyzer 2100 and High Sensitivity DNA Kit (Aligent). Barcoded RNA sequencing (RNA-seq) libraries were pooled and sequenced together on the Ion Torrent S5 sequencer by using P1 chips. Sequencing reads were mapped to AmpliSeq transcriptome target regions with the torrent mapping alignment program and quantified with the Ion Torrent AmpliSeq RNA plugin using the unique mapping option.

### Single-cell RNA sequencing

One sample used in targeted bulk RNA-seq was split and co-prepared for sc-RNA-sequencing. ScRNA-seq was performed using the Chromium Single Cell 3’ Library and Gel Bead Kit v3 (10x Genomics) using the Chromium Controller according to the manufacturer’s protocol. Single cell libraries were sequenced on an Illumina NovaSeq 6000.

### Single-cell RNA-seq analysis

Demultiplexing, alignment to the mm10 transcriptome and unique molecular identifier (UMI)-collapsing were performed using the Cell Ranger toolkit (version 2.1.0, 10x Genomics). All post-quantification quality control and analysis was performed using the Seurat analysis suite [108]. For each cell, we quantified four library/sequencing quality metrics: number of genes for which at least one read was mapped, total number of UMIs, percent of total UMIs which mapped to mitochondrial genes, and percent of total UMIs which mapped to ribosomal genes. All cells which had between 500 and 4500 unique expressed genes, between 1000 and 30000 UMIs, less than 5% mitochondrial UMIs (to exclude lysed cells), and greater than 20% ribosomal UMIs (to exclude mitochondrial/ribosomal outliers) were used for downstream analysis. Expression values *Ei*, *j* for gene *i* in cell *j* were calculated by dividing UMI count values for gene *i* by the sum of the UMI counts in cell *j*, to normalize for differences in coverage, then multiplied by 10,000 to create transcript per million (TPM)-like values, and finally calculating log2(TPM+1) values.

The gene set analyzed was further restricted to those with the highest variability based on scaled non-zero mean expression greater than 0.05 and log(variance-to-mean ratio) greater than 1.0. Expression values were centered and scaled before input to principal component analysis (PCA). Using the elbow principle, the first 7 principal components (PCs) were selected for input to t-SNE. For visualization purposes, dimensionality was further reduced using the Barnes–Hut approximate version of t-SNE with a perplexity of 50. The first 7 PCs were separately used to generate expression-based cell clusters using the Louvain algorithm with clustering resolution = 0.4. To identify differentially expressed genes in the single cell data we performed pairwise Wilcoxon tests.

### Trajectory analysis

Demultiplexing, alignment to the mm10 transcriptome, and UMI-collapsing was performed using the Cellranger toolkit (version 3.1, 10X Genomics). Monocle 3 (Trapnell Lab) was used to filter, normalize, reduce dimensions, cluster, and perform trajectory and pseudotime analysis. Detect genes was set to a minimum threshold of 0.1 and only cells that expressed between 750–3000 genes were used for downstream analysis. Further quality metrics limited analysis to distribution of UMI across cells to those within 2 standard deviations from mean mRNA count. UMAP non-linear dimension reduction was performed on data pre-processed in 15 dimensions followed by clustering using the Louvain hierarchical clustering algorithm. This data was then used as the input for trajectory and pseudotime analysis within the Monocle 3 program where starting point was chosen based on central cluster that contained cells of both lymph node and lung origin and Tfh (Id3) and Th2 (Gata3) expression.

### ATAC-sequencing

Preparation of cells, libraries, and sequencing was performed as previously described [109]. Briefly, IL-4^4get^ and IFNγ^GREAT^, C57BL/6 mice were infected with *N*. *brasiliensis*, the lung and mediastinal lymph nodes were harvested eight days post-infection, and single cell suspensions were prepared as described. IL-4-competent and IFN-gamma-competent CD4^+^ T cells (DAPI-negative, CD3ε^+^, CD4^+^, CD11b^-^, CD11c^-^, CD8^-^, B220^-^, NK1.1^-^, Ter119^-^, GFP^+^/YFP^+^) and cytokine-negative (GFP^-^/YFP^-^) CD4^+^ T cells were sorted. 50,000 cells from each sample were washed in cold PBS, lysed, and transposed for 40 minutes at 37°C using Nextera Tn5 transposase enzyme (Illumina). Transposed DNA fragments were amplified for 10–15 cycles and barcoded using Illumina compatible indexed primers purchased from integrated DNA Technologies (https://IDTdna.com). Library purification was performed with the MinElute PCR Purification Kit (Qiagen) and size selection was using AMPureXP beads (Beckman Coulter). Libraries were quantified and size distribution was assessed using the Bioanalyzer High Sensitivity DNA Kit (Agilent). Paired-end sequencing was performed on a HiSeq 2500 (Illumina) with 50 cycles for each read.

### ATAC-seq analysis

Raw data from the sequencer was demultiplexed and FASTQ files were generated using bcl2fastq Conversion Software (Illumina). Nextera adapters were trimmed using skewer (version 0.2.2) before mapping to the mouse genome (GRCm38/mm10) using bowtie (version 2.3.2 with options—very-sensitive—no-dovetail -X 2000) [110,111]. Potential PCR duplicates were marked using Picard Tools (version 2.8.1, https://broadinstitute.github.io/picard/). Any reads that were either umapped, not in proper pairs, had a mapping quality score of less than 10, or mapped to the mitochondrial chromosome, non-canonical contigs, or the ENCODE3 blacklisted regions (accession ENCFF759PJK) were removed. Peak summits were identified from individual replicates using MACS2 (version 2.1.1, March 9, 2016, with parameters—keep-dup auto—nomodel—nolambda). Regions of uniform width (200 nt) centered on the peak summits were generated from each replicate and overlapping regions between replicates were merged. Peaks on the Y chromosome, as well as within ENCODE blacklisted regions (accession ENCFF547MET) were excluded from further analysis. Differential coverage comparison analysis was performed as described elsewhere [112]. The number of transposase insertions within each region was computed for each replicate with summarizeOverlaps from the BioConductor package GenomicAlignments (verion 1.14.2) and normalized with voom from the limma package (version 3.38.3) in R (version 3.5.1). Pairwise contrasts were performed with limma and differentially accessible regions were filtered based on an FDR-adjusted p-value of less than 0.05 and an estimated fold-change of at least 3. We computed ATAC-seq density (number of transposase insertion sites per kilobase per million insertions) per peak and accessible regions were defined as those with a mean of 5 normalized insertions per kilobase. HOMER (v4.10.4) was used to identify enriched transcription factor motifs and their genomic location using the GRCm38/mm10 assembly of the mouse genome.

### Statistical analysis

Data are presented as the mean ± standard deviation (SD) from the specified number of mice unless otherwise noted. Statistical calculations were performed with Prism 7.0 software (GraphPad). Comparison of data between two groups was analyzed using a two-tailed unpaired *t* test to determine statistical significance. In the figures indicated, N.S. designates non-significant statistical differences. In all figures, only statistical differences between WT and experimental groups in either naïve, infected, or treated groups are displayed. Where possible exact p-values are provided in figures.

## Supporting information

S1 Fig Associated with Fig 1. IL-4-expressing CD4^+^ T cells in the mediastinal lymph node and lung in the same mouse share TCRβ sequences but few public clones are shared across mice. IL-4^4get^ C57BL/6 or BALB/c mice were infected with *N*. *brasiliensis* and GFP^+^ CD4^+^ T cells from the lung and mediastinal lymph nodes were harvested and GFP^+^ IL-4-competent CD4^+^ T cells were sorted nine days post-infection for TCRβ analysis. **(A-C)** Diagonal scatter plots comparing Log_2_-normalized TCRβ CDR3 amino acid or nucleotide sequences of IL-4-expressing CD4^+^ T cells from indicated mice/genetic background. Red boxes indicate intramouse comparisons. **(D)** Jaccard similarity coefficient matrix comparing the similarity of TCR-β sequences found in the mediastinal lymph nodes and lungs of different BALB/c IL-4^4get^ mice and the lymph nodes and lung of the same mouse. Red boxes indicate intramouse comparisons. **(E)** The graphs represent the percentage of TCRβ sequences found at least 5 times in one tissue or represent the top 10 most abundant TCRβ sequences located in the indicated tissues that are shared within the same BALB/c IL-4^4get^ mouse or across different mice. Error bars represent +/- SD; n = 3 mice.
(TIF)

S2 Fig Associated with Fig 5. Distribution of HCAF-GLIPH consensus clusters in scTCR repertoire of IL-4^+^ CD4^+^ T cells after helminth infection. **(A)** Table represents the HCAF-GLIPH consensus clusters identified in the scTCR dataset. The size and name of each HCAF and GLIPH cluster used to generate the consensus cluster is identified. The relative percentage of the sequences in each cluster belonging to specific or shared tissues or mice is provided. **(B)** Diagrams of HCAF-GLIPH consensus clusters outlined in (A). Oval represents sequences found in GLIPH cluster and rectangle represents sequences found in the HCAF cluster. Where the oval and rectangle overlap is the consensus cluster. The consensus amino acid sequence of the Vβ region in each consensus cluster is provided.
(TIF)

S3 Fig Associated with Fig 6. Gating scheme for sorting IL-4^+^ and IL-4^-^ CD4^+^ T cells from the lung and mediastinal lymph nodes for bulk and scRNA-sequencing. IL-4^4get^ C57BL/6 mice were infected with *N*. *brasiliensis* and GFP^+^ and GFP^-^ CD4^+^ T cells from the mediastinal lymph nodes (A, B) and lung (C, D) were harvested and sorted nine days post-infection for bulk RNA AmpliSeq and scRNA-seq analysis. **(A)** Pre-sort gating scheme for the mediastinal lymph node used for paired bulk and scRNA-sequencing. **(B)** Contour plots depict purity of ungated cells sorted from (A) used for library preparations. **(C)** Pre-sort gating scheme for the lung used for paired bulk and scRNA-sequencing. **(D)** Contour plots depict purity of ungated cells sorted from (C) used for library preparations. Representative of 6 bulk AmpliSeq and 1 scRNA-seq experiment.
(TIF)

S1 Table Associated with Fig 2. Sharing of top 10 clones among IL-4^-^, mRNA^+^, and protein^+^ T cells with other groupings. Table shows information regarding the TCR sequence and family for each of the top 10 clones (by count) observed after single cell TCR sequencing. In addition, the table shows the number of times a top 10 clone is observed within other groupings. Different highlighted colors indicate when a top 10 clone is shared among the top 10 clones of other groups.
(TIF)
