# Supplementary figures and images for: Single cell analysis of host response to helminth infection reveals the clonal breadth, heterogeneity, and tissue-specific programming of the responding CD4+ T cell repertoire
Source: PLoS Pathog. 2021 Jun 9;17(6):e1009602. doi: 10.1371/journal.ppat.1009602 (PMC8216541; doi:10.1371/journal.ppat.1009602)

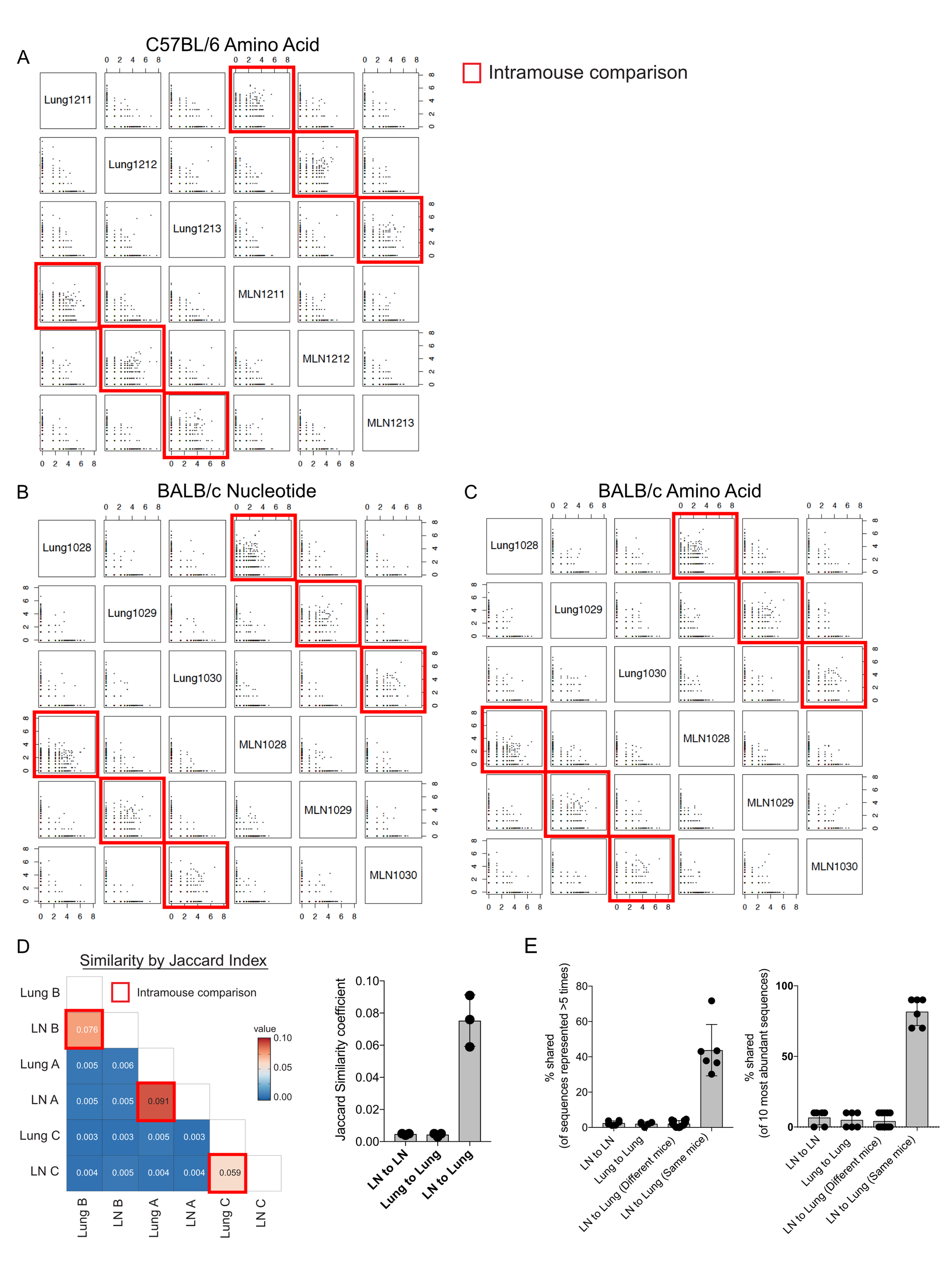

Supplement: S1 Fig — IL-44get C57BL/6 or BALB/c mice were infected with N. brasiliensis and GFP+ CD4+ T cells from the lung and mediastinal lymph nodes were harvested and GFP+ IL-4-competent CD4+ T cells were sorted nine days post-infection for TCRβ analysis. (A-C) Diagonal scatter plots comparing Log2-normalized TCRβ CDR3 amino acid or nucleotide sequences of IL-4-expressing CD4+ T cells from indicated mice/genetic background. Red boxes indicate intramouse comparisons. (D) Jaccard similarity coefficient matrix comparing the similarity of TCR-β sequences found in the mediastinal lymph nodes and lungs of different BALB/c IL-44get mice and the lymph nodes and lung of the same mouse. Red boxes indicate intramouse comparisons. (E) The graphs represent the percentage of TCRβ sequences found at least 5 times in one tissue or represent the top 10 most abundant TCRβ sequences located in the indicated tissues that are shared within the same BALB/c IL-44get mouse or across different mice. Error bars represent +/- SD; n = 3 mice. (TIF) [file ppat.1009602.s001.tif]

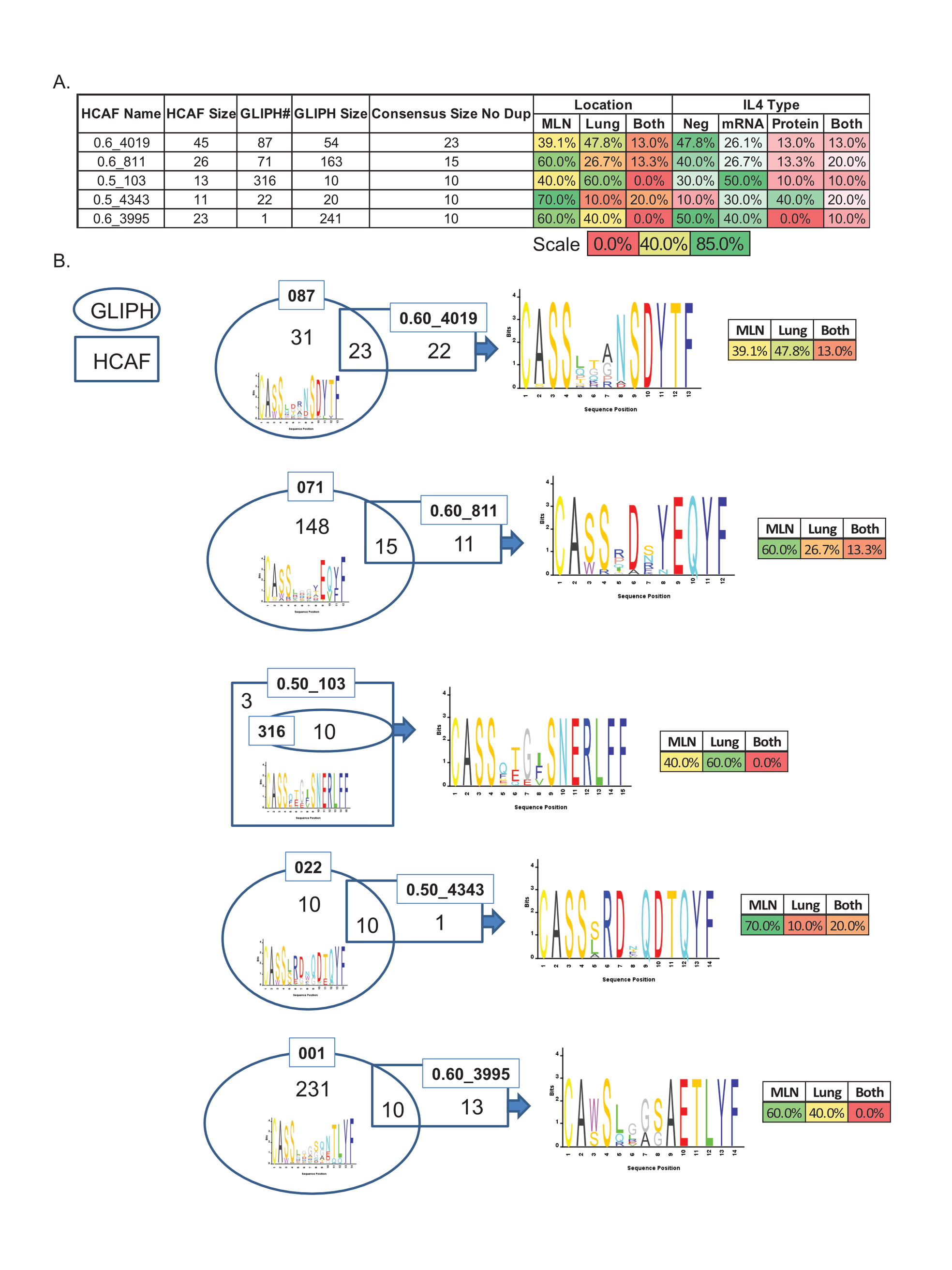

Supplement: S2 Fig — (A) Table represents the HCAF-GLIPH consensus clusters identified in the scTCR dataset. The size and name of each HCAF and GLIPH cluster used to generate the consensus cluster is identified. The relative percentage of the sequences in each cluster belonging to specific or shared tissues or mice is provided. (B) Diagrams of HCAF-GLIPH consensus clusters outlined in (A). Oval represents sequences found in GLIPH cluster and rectangle represents sequences found in the HCAF cluster. Where the oval and rectangle overlap is the consensus cluster. The consensus amino acid sequence of the Vβ region in each consensus cluster is provided. (TIF) [file ppat.1009602.s002.tif]

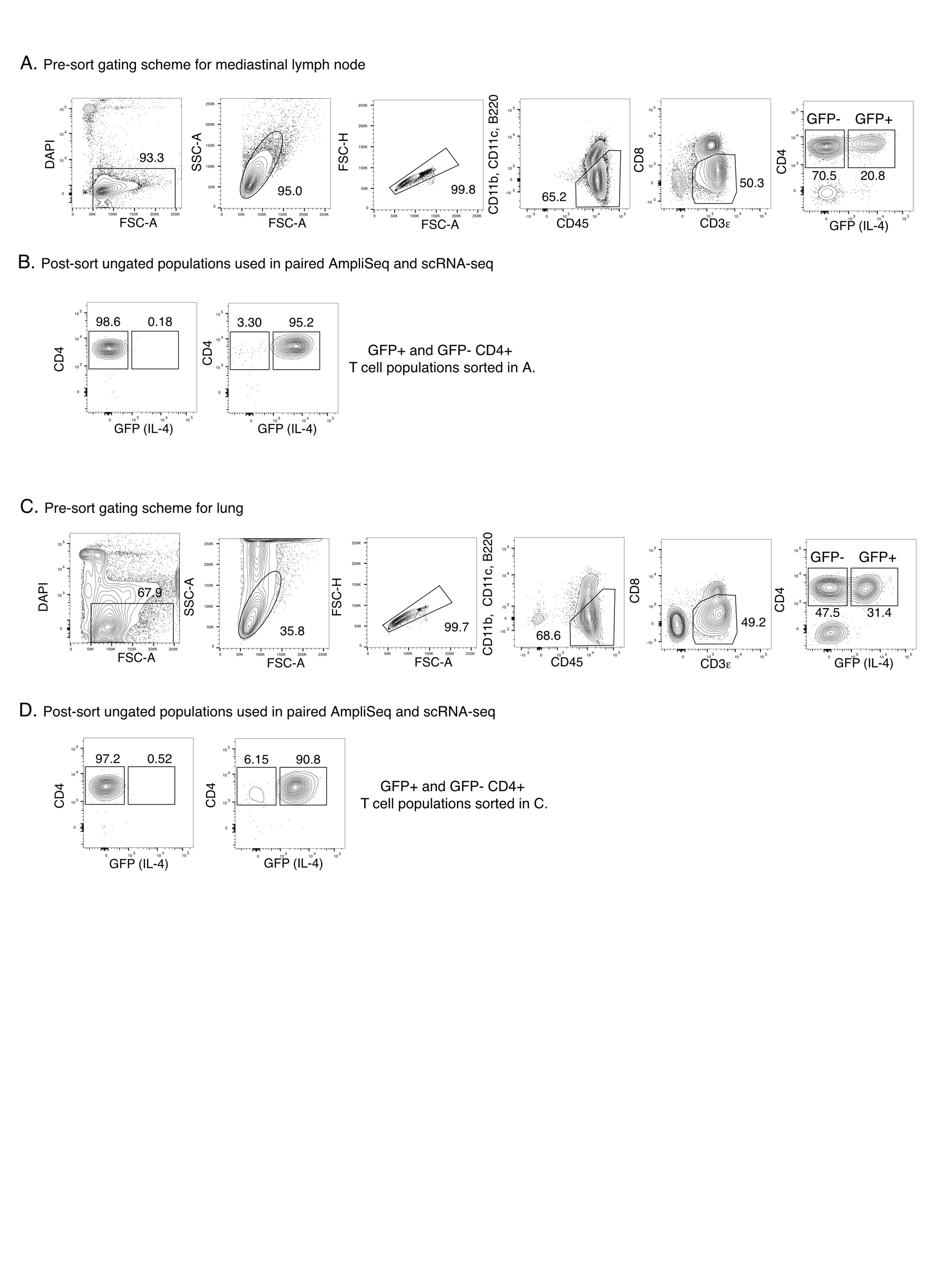

Supplement: S3 Fig — IL-44get C57BL/6 mice were infected with N. brasiliensis and GFP+ and GFP- CD4+ T cells from the mediastinal lymph nodes (A, B) and lung (C, D) were harvested and sorted nine days post-infection for bulk RNA AmpliSeq and scRNA-seq analysis. (A) Pre-sort gating scheme for the mediastinal lymph node used for paired bulk and scRNA-sequencing. (B) Contour plots depict purity of ungated cells sorted from (A) used for library preparations. (C) Pre-sort gating scheme for the lung used for paired bulk and scRNA-sequencing. (D) Contour plots depict purity of ungated cells sorted from (C) used for library preparations. Representative of 6 bulk AmpliSeq and 1 scRNA-seq experiment. (TIF) [file ppat.1009602.s003.tif]

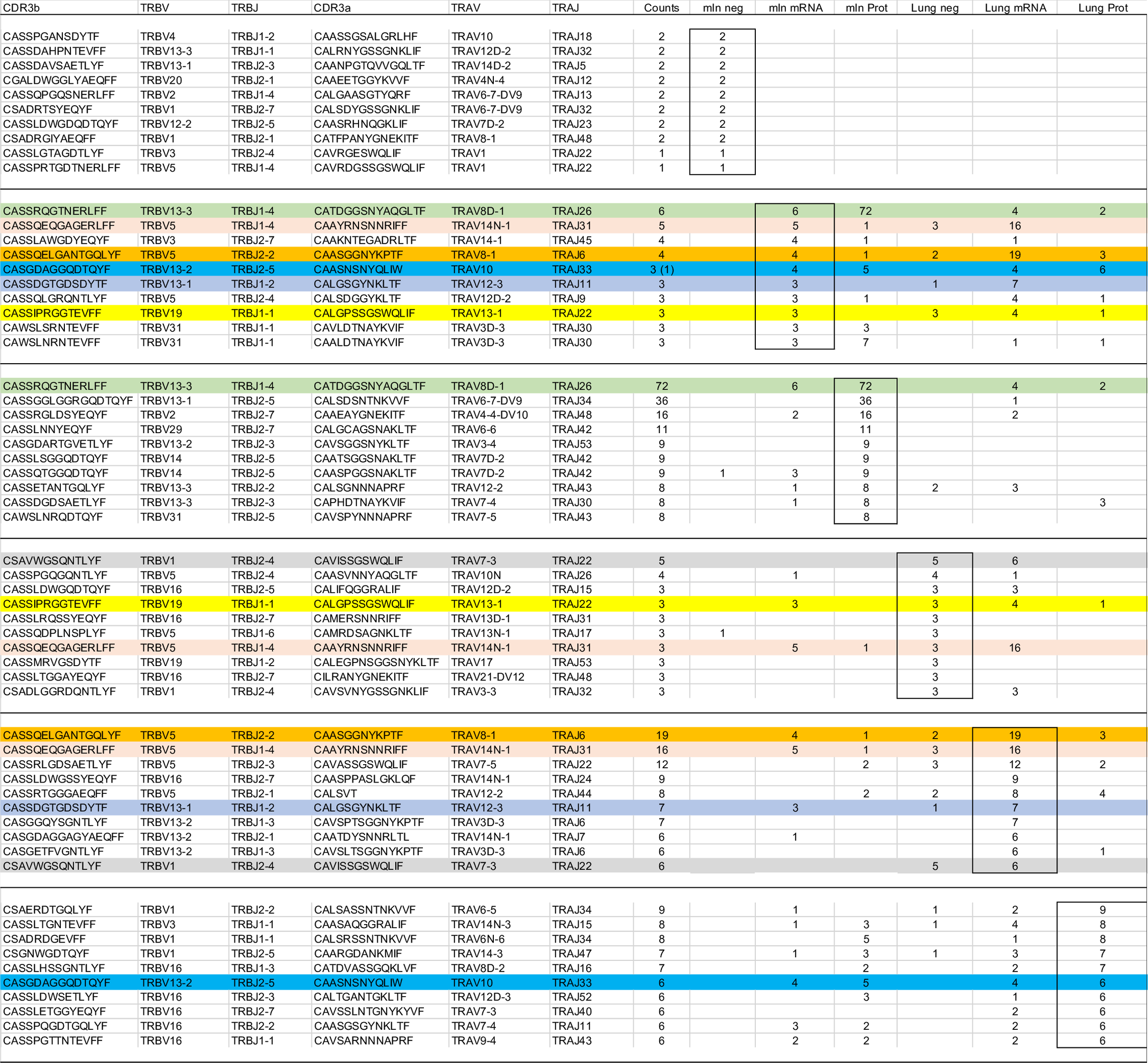

Supplement: S1 Table — Table shows information regarding the TCR sequence and family for each of the top 10 clones (by count) observed after single cell TCR sequencing. In addition, the table shows the number of times a top 10 clone is observed within other groupings. Different highlighted colors indicate when a top 10 clone is shared among the top 10 clones of other groups. (TIF) [file ppat.1009602.s004.tif]
